# Reassembling a cannon in the DNA defense arsenal: Genetics of StySA, a BREX phage exclusion system in *Salmonella* lab strains

**DOI:** 10.1371/journal.pgen.1009943

**Published:** 2022-04-04

**Authors:** Julie Zaworski, Oyut Dagva, Julius Brandt, Chloé Baum, Laurence Ettwiller, Alexey Fomenkov, Elisabeth A. Raleigh

**Affiliations:** Research Department, New England Biolabs, Ipswich, Massachusetts, United States of America; Universidad de Sevilla, SPAIN

## Abstract

Understanding mechanisms that shape horizontal exchange in prokaryotes is a key problem in biology. A major limit on DNA entry is imposed by restriction-modification (RM) processes that depend on the pattern of DNA modification at host-specified sites. In classical RM, endonucleolytic DNA cleavage follows detection of unprotected sites on entering DNA. Recent investigation has uncovered BREX (BacteRiophage EXclusion) systems. These RM-like activities employ host protection by DNA modification, but immediate replication arrest occurs without evident of nuclease action on unmodified phage DNA. Here we show that the historical *stySA* RM locus of *Salmonella enterica* sv Typhimurium is a variant BREX system. A laboratory strain disabled for both the restriction and methylation activity of StySA nevertheless has wild type sequence in *pglX*, the modification gene homolog. Instead, flanking genes *pglZ* and *brxC* each carry multiple mutations (μ) in their C-terminal domains. We further investigate this system *in situ*, replacing the mutated *pglZμ* and *brxCμ* genes with the WT counterpart. PglZ-WT supports methylation in the presence of either BrxCμ or BrxC-WT but not in the presence of a deletion/insertion allele, Δ*brxC*::*cat*. Restriction requires both BrxC-WT and PglZ-WT, implicating the BrxC C-terminus specifically in restriction activity. These results suggests that while BrxC, PglZ and PglX are principal components of the BREX modification activity, BrxL is required for restriction only. Furthermore, we show that a partial disruption of *brxL* disrupts transcription globally.

## Author summary

Horizontal gene transfer is a major driver of evolution and adaptation in bacteria. Genes from outside may be beneficial or dangerous to the receiving cell. Benefits include new food sources such as sugars, or new homes by adhesion, or new resistances, as to antibiotics. Dangers are posed by bacteriophages--viruses that take over the cell machinery, multiply, and release progeny to kill sister cells. Host-dependent restriction-modification systems enable defense that distinguishes relatives from strangers: using a modification pattern (M) carried by DNA bases added by the host cell to prevent restriction (R). Sisters and cousin cells will have the same protective pattern on DNA, while DNA of foreign origin will have the wrong M pattern and be restricted (R, rejected). Typically, restriction involves nuclease digestion. Here we address the enigmatic StySA RM system, one of the earliest to be genetically characterized. It is a variant of the newly recognized defense mechanism, BREX. BREX systems also track DNA history via modification pattern, but restrict by a novel, uncharacterized mechanism. Like other BREX family systems, StySA-BREX modification requires multiple components. When StySA-BREX transcription is unbalanced, we find global disruption of gene transcription. The disruption pattern does not suggest SOS-inducing damage to DNA.

## Introduction

Transfer of genes from one organism to another shapes ecological capacities in microbiomes on both short and long-term timescales. Thus, mechanisms that limit or promote such transfer are of fundamental interest. Ecologic interactions with phage play a major role in host colonization by prokaryotes [1,2]. Prokaryote defenses against phages are of particular interest [3,4], particularly as therapeutic uses are contemplated [5]. Microbiome and metagenome studies have led to a renaissance in the study of phage-host interaction [6,7].

Host defenses include restriction-modification (RM) systems as major contributors [8]. These distinguish the host DNA from foreign invaders using the pattern of DNA modification: DNA with the wrong modification pattern is rejected [9–11]. Classical restriction endonucleases cleave both DNA strands in response to the presence of unmethylated specific sites, while protection is conferred by DNA methylation at adenine or cytosine residues within the specific site. RM-like systems are also known in which nuclease action is prevented by sequence-dependent sulfur modification of the DNA backbone [12]. DNA cleavage leads to very rapid interruption of the phage development program.

Both defensive and epigenetic processes can involve DNA modification states, so taxa with no DNA modification are extremely rare. Epigenetic regulation is important in the life of the cell, and often the relevant genes are fixed in a lineage [13,14]. In contrast, defense functions are diverse, often clustered in variable "defense islands" [6,15–17]. These specialized "defense islands" are enriched in genes that specifically regulate DNA entry [8,18–22]. Defenses include RM systems (including modification-dependent nucleases, MDRS) [1], and a wide variety of "abortive infection" elements, which protect siblings but not the infected cell [3,23,24]. A novel modification-dependent defense system, Pgl (phage growth limitation), was studied by M.C. Smith and coworkers [25–27] in *Streptomyces coelicolor*. Unusually, the host genome is *not* methylated, while progeny phage *are* methylated at sites specified by PglX. Modification renders the phage susceptible to subsequent restriction by MDRS upon infection of sibling cells.

Sorek and co-workers extended the suite of methylation-protected defense systems, using neighborhood analysis anchored by homologs of a component of the Pgl system, PglZ [28,29]. This identified a set of systems designated BREX (Bacteriophage Exclusion), gene clusters of 4 to 8 genes, depending on the subtype. A BREX system from *Bacillus cereus* was studied experimentally in *B*. *subtilis* by Goldfarb et al. [28]. Examples of BREX system have also been found in other bacteria [17,30–32]. Though some components of BREX are related to Pgl, the two families displayed important differences in biological endpoints, particularly the role of methylation, which protects BREX hosts [30] but elicits restriction by Pgl^+^ sibling cells. BREX does not appear to restrict by cleaving DNA in vivo [28].

SenLT2III (StySA) is one of three classical RM systems in *S*. *enterica* sv Typhimurium LT2. The other two are multicomponent ATP-dependent systems of Type III (SenLT2I; LT, StyLT in the early literature) and Type I (SenLT2II; SB, StySB). For clarity, here we use StySA below for the RM system under study. StySA was shown to modify adenine residues [33] and resembled Type I enzymes in sensitivity to by anti-restriction activities of T7 OCR [34] and P1 DarA [35]. However, DNA cleavage was never demonstrated. We identified the genomic location of the StySA system while analyzing the sequences of *S*. Typhimurium-*S*. Abony hybrid strains [36]. Notably, this is within a variable chromosomal island anchored by *leuX* where numerous non-homologous mobile elements are found in *E*. *coli* and *Salmonella* [37].

In this study, we identify the StySA RM system as a variant BREX system and report the genetic contributions of its constituent genes to host protection and interruption of phage infection. The transcriptional organization of the locus contributes to understanding of relative transcription levels in the native context. We find that *brxC* and *pglZ* mutations in ER3625 are responsible for the lack of restriction and modification; only the N-terminal domain of BrxC is required for modification. Serendipitously, we present evidence that the BrxL C-terminal domain by itself can have a large effect on host gene transcription, particularly a non-SOS effect on resident prophages.

## Results

### Strain engineering design: Domains, mutation clusters and transcription start sites

#### StySA is a BREX variant

Our experimental host, ER3625, is a descendant of the model organism LT2 carrying a mutated allele of StySA with other known RM systems disabled. Automated annotation of its genome [36,38] predicted a relationship of the StySA region of ER3625 to BREX and Pgl systems. We adopt the automated name assignments from NCBI; to facilitate reference to the ancestor LT2, at the first appearance of each gene we include the LT2 Locus_ID in parentheses. The invariant Locus_IDs and Protein_IDs in database records are listed in S1 Table for the BREX systems of *E*. *coli* HS, *S*. Typhimurium LT2 and experimental host ER3625.

**Fig 1:**
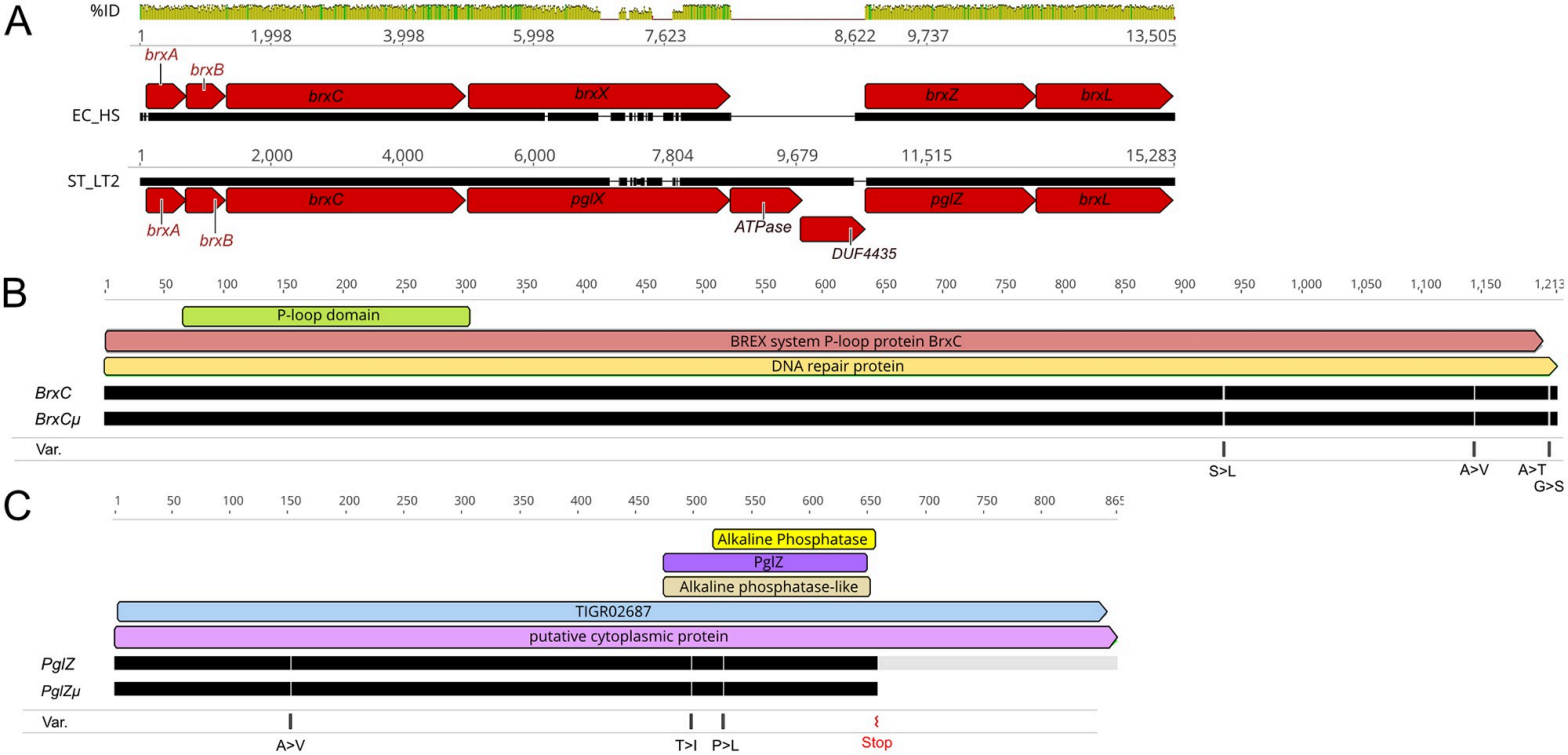
StySA locus similarity to *E*. *coli* BREX DNA sequence and protein variants in ER3625. Panel A: DNA alignment of BREX regions from LT2 and *E*. *coli* HS. %ID: dark green 100% identity, light green, close similarity, white: missing regions. Numbers: nt coordinates of segments extracted from the genome sequences (EC_HS, ST_LT2). Red arrows: annotated BREX genes; black segments: aligned nt, with breaks where the two don’t match. Panels B and C: Domain assignments and protein alignments for BrxC (LT2) and BrxCμ (ER3625), panel B; and PglZ (LT2) and PglZμ (ER3625), panel C. Colored boxes: domain predictions; black lines: aligned aa sequences with breaks at variant positions and a grey extension for the LT2 PglZ C-terminal region after the stop codon in ER3625; Var: amino acids changes found in ER3625.

DNA alignment of BREX locus of LT2 [39] and the characterized *E*. *coli* HS BREX locus [30] yields about 80% sequence identity, interrupted by a large indel between *pglX* (STM4495) and *pglZ* (STM4492; Fig 1). The *S*. Typhimurium BREX variant contains a two-gene region that is missing in the *E*.*coli* HS variant. These are annotated as ATPase (STM4493) and DUF4435 (STM4494). Neither gene contributes to the modification activity *in vivo* (see below "Phenotypic consequences of strain engineering"). Conversely, a short region just upstream of *E*. *coli* HS *brxZ* is missing in the *Salmonella* LT2 copy. This may correspond to a promoter/regulatory region for *brxZ/pglZ in E*. *coli*. In LT2, a promoter in DUF4435 apparently provides transcription (see below, "Transcription overview").

ER3625 carried the DNA modification pattern expected for an LT2 derivative except for StySA sites (GATC**A**G, modification on the bold A), which were not modified [36]. The expected MTase, *pglX*, has no changes from LT2. Instead, two flanking genes corresponding to *pglZ* and *brxC* (STM4496) did vary from the LT2 sequence.

The characterized Pgl system expresses a putative phosphatase (*pglZ*), a protein kinase (*pglW*), a candidate adenine-specific DNA methyltransferase (*pglX*) and a P-loop ATPase (*pglY*) [25]. Proteins specified by two of the 6 genes in type I BREX are related: PglZ and PglX. Additional BREX-specific genes are *brxA* (STM4498), proposed to be a structural homologue of RNA-binding antitermination protein NusB; *brxB* (STM4497), coding a protein of unknown function; *brxC*, coding a large protein with a P-loop ATP-binding domain (sometimes identified as PglY); and *brxL* (STM4491), identified as a Lon-like protease-domain [28].

Schematic domain predictions are shown in Fig 1B for the relationship between the ancestral LT2 and derived ER3625 proteins: BrxC of LT2 and BrxCμ of ER3625; Fig 1C PglZ (LT2) and PglZμ (ER3625). Annotation was carried out as described in S6 File.

ER3625 genes descended from *brxC* and *pglZ* will be designated *brxCμ* (coding for BrxCμ) and *pglZμ* (coding for PglZμ) respectively. Each carries multiple mutations relative to the LT2 ancestor. The amino-acid changes resulting from these are displayed in Fig 1B (BrxC) and Fig 1C (PglZ).

#### Genetic approach to the StySA locus

We chose to investigate this system *in situ*, replacing the mutated *pglZμ* and *brxCμ* genes with ancestral LT2 sequence. The strategy for gene deletion and replacement employed a method that leaves no scars, via an intermediate carrying a drug resistance cassette with its own promoter (see Materials and methods). The engineering strategy first required a sketch of possible transcription signals in LT2 (strain Z of Table 1). We wished to understand how transcription is organized, and to avoid removing promoters or termination signals as much as possible. Segments chosen for engineering (S1 Fig) used primers designed from these results (S1 File).

**Table 1:** Strain names, labels, and phenotype summary.

Strain ID	Strain number	Short genotypes	Phenotype label^a^	StySA methyl-ation^b^ %	Dam methyl-ation^b^ %	R^c^ mag-nitude	Growth^d^
A	ER3625	*Zμ Cμ*	R^-^M^-^	1.1	97	< 10	normal
B	JZ_022	*Z+ Cμ*	R^-^M^+^	94.7	99	< 10	normal
C	JZ_028	*Zμ C+*	R^-^M^-^	0.2	99.7	< 10	slow
D	JZ_040	*Z+ CΔ*	R^-^M^-^	5.7	99.8	< 10	normal
E	JZ_043	*ZΔ C+*	R^-^M^-^	1	99.8	< 10	normal
F	JZ_052	*Z+ Cμ ΔL*	R^-^M^+^	99	99.6	< 10	normal
G	JZ_055	*Z+ Cμ ΔB*	R-M^p^	46.2	99.9	< 10	very slow
H	JZ_056	*Z+ Cμ ΔB*	R^-^M^p^	65.6	99.9	< 10	very slow
I	JZ_057	*Z+ Cμ ΔB*	R^-^M^p^	36.2	99.8	< 10	very slow
J	JZ_058	*Z+ C+*	R^+^M^+^	99.2	98.9	> 100	normal
K	JZ_061	*Zμ C+ ΔB*	R^-^M^+^	83.4	99.9	< 10	very slow
L	JZ_064	*Zμ C+ ΔL*	R^-^M^+^	1.1	99.9	< 10	normal
M	JZ_069	*Z+ C+ ΔATPase*	R^?^M^+^	99.3	99.5	?	slow
O	JZ_072	*Z+ C+ ΔB*	R^?^M^-^	97.5	97	?	very slow
P	JZ_074	*Z+ C+ ΔL*	R^?^M^+^	100	99.9	?	very slow
Q	JZ_080	*Z+ C+ ΔDUF4435*	R^?^M^+^	99.9	99.9	?	slow
R	JZ_091	*Z+ Cμ ΔDUF4435*	R^-^M^+^	97.1	99.9	< 10	slow
S	JZ_092	*Z+ Cμ ΔATPase*	R^-^M^+^	99.1	99.8	< 10	normal
T	JZ_094	*Zμ C+ ΔDUF4435*	R^-^M^+^	0.4	99.9	< 10	normal
U	JZ_095	*Zμ C+ ΔATPase*	R^-^M^+^	0.5	100	< 10	slow
V	JZ_105	*Z+ Cμ Δ(mrr2)*	R^-^M^+^	99.6	99.9	< 10	normal
W	JZ_106	*Zμ C+ Δ(mrr2)*	R^-^M^-^	0.5	99.9	< 10	normal
X	OD_127	*Δ(Z-C)*	R^-^M^-^	0	99.7	< 10	normal
Y	ER3649	*Z+ C+*	R^+^M^+^	-	-	> 100	normal
Z	LT2 (STK013)	*Z+ C+*	-	94.4	96	-	-
AA	STK005	*Zμ Cμ*	R^-^M^-^	[36]	[36]	-	-
AB	JB_009	*ΔStySA*	R^-^M^-^	0	99	< 10	normal

^a^R^-^ non-restricting; R^+^ restricts phage L; R^?^ resistant to phage L and broth cultures settle; M^-^ StySA modification severely deficient; M^+^ StySA modification normal; M^p^ StySA sites partially modified. "-" not tested by us.

^b^Fraction of sites with N6mA at GATCAG or GATC in genomic DNA measured with Pacific Biosciences method (see Materials and methods). "-" not tested. Strains assigned M^-^ display modification of <6% of StySA sites; M^p^ 36–65% modified; M^+^ >83% modified

^c^StySA-unmodified phage L spot titer ratio (ER3625/strain X). ER3649 is the restricting control. "?" not testable, "-" not tested.

^d^Growth rate in deepwell plates; see text. "-" not tested.

#### Transcription overview: WT StySA transcripts with Cappable-Seq and RNAseq

**Fig 2:**
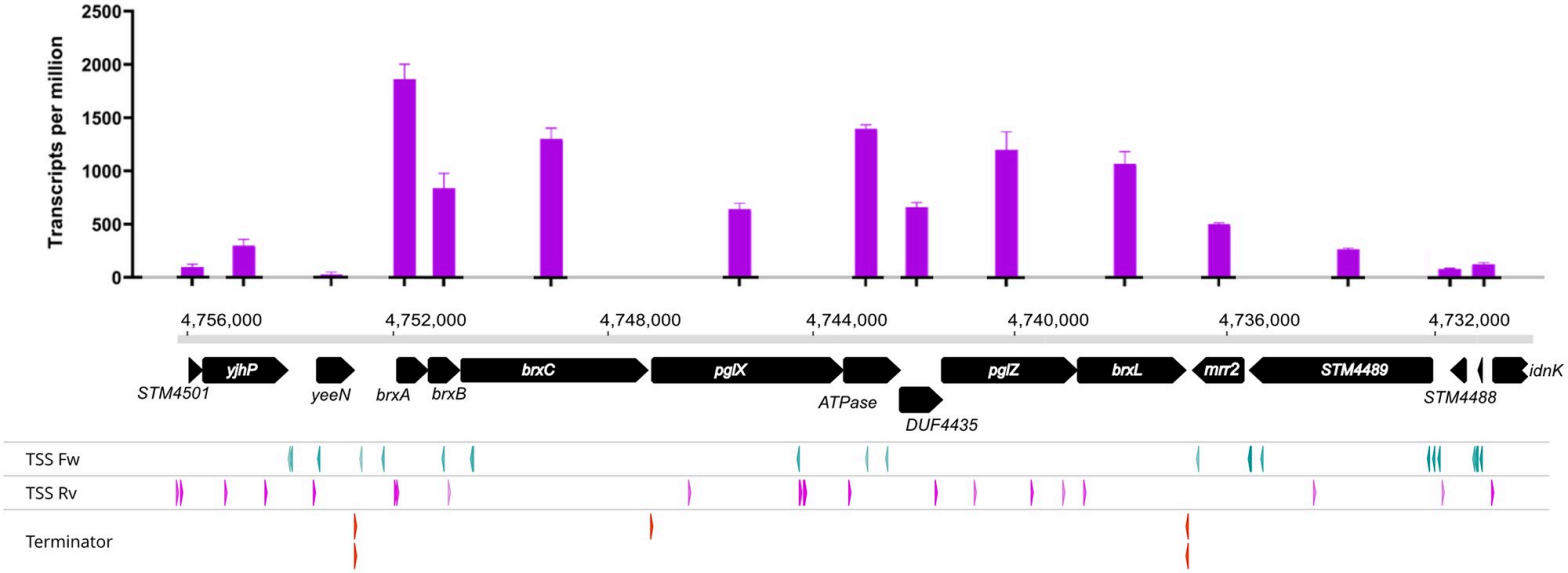
StySA locus operon structure analysis. The top graph represents the transcript level for each annotated CDS. Y-axis coordinates are TPM; X-axis nucleotide coordinates are from LT2 sequence NC_003197.2 (note that nt coordinates read right to left; this orients most CDS left to right). Black arrows are CDS (to scale), labelled with the BREX nomenclature of Fig 1. The BREX-related cluster comprises *brxA-brxL*; on the flanks, three (left) and five (right) external CDS are included to assess transcription into or out of the locus. Below the gene schema, transcription start sites (TSS) are presented. "Fw" TSS are top strand starts for NC_003197.2, "Rv" are bottom strand starts, including BREX promoters. Rho-independent transcription terminators were predicted using TransTerm.

We investigated transcription start sites in the ancestral LT2 (strain Z) with Cappable-seq [40] and RNAseq data from our derived strain with WT sequence (*Z*^*+*^*C*^*+*^ JZ_058, strain J) to relate transcript abundance to our mapped TSS and bioinformatically predicted terminators (Fig 2 and S10 File).

With Cappable-seq, two biological replicates identified 15,650 and 15,145 unique TSS positions in the genome (Materials and methods) (above the TPM > = 1.0 and EnrichRatio > = 2.5). Clustering was used to regroup close TSS giving a final count of 9,422 and 9,777 TSS clusters. Of these, 8,041 are shared positions with an adjusted R2 = 0.96426 and a P-value < 2.2E^-16^. Whole genome TSS data can be found at https://www.ncbi.nlm.nih.gov/sra/?term=SAMN17272070 in NCBI.

With this highly accurate method for mapping TSS location genome-wide, we confirm in our LT2 isolate (STK013) some TSS already made publicly available [41] and identify some new intragenic TSS (Fig 2). Primary TSS upstream of *brxA* and within *DUF4435* are confirmed; internal TSS are found within *pglX*, and *ATPase*. A strong TSS cluster is located upstream of *mrr2* oriented toward the BREX cluster but outside it. Additional antisense TSS can also be found.

Analysis of RNASeq data allows us to quantify individual gene expression. Fig 2 displays this for strain J (LT2 sequence replaces that of ER3625 in this strain). Transcript abundance for each feature can be related to TSS (pink arrows in Fig 2; green arrows are mostly the antisense strand) and predicted terminators (bottom row).

Rho-independent terminators are predicted at three positions in this locus. Two are bidirectional, flanking the StySA locus--before *brxA* and after *brxL*. The locus should thus be transcriptionally insulated (see "Local and global inferences from transcriptome profiling" belo**w**).

Experiments combining long-read reverse transcription selective for 5’ triphosphates with 3’ poly-A addition [42] to acquire transcript ends (SMRT-Cappable-seq-RACE) suggest that *brxA*, *B* and *C* and *pglX* are on one transcript (S8 File panel A), while *ATPase*, *DUF4435*, *pglZ* and most likely *brxL* are on at least one additional transcript (S8 File panel D). TSS within *ATPase* and *DUF4435* are both validated. Results for the *brxA* TSS suggest incomplete termination at the third predicted terminator, between *brxC* and *pglX* (S8 File panel A): read number drops at the end of *brxC* (S8 File panels B and C), with some read-through to *brxX*. Higher coverage would be needed to support stronger conclusions (~15 reads before the terminator position and ~5 after it).

#### Phenotypic consequences of genome engineering

23 strains created for this work and five control hosts are listed in Table 1 and S1 File. For each constructed strain and ER3625, four phenotypic measurements were carried out: phage restriction; modification at StySA sites (using Dam modification as a control); growth rate; transcript level of the MTase candidate (*pglX*). For a selection of strains, RNAseq was used to analyze local and global transcription effects.

#### PglZμ and BrxCμ contribute to both R^-^ and M^-^ properties

Of all the engineered strains, only one R^+^ strain was obtained (Table 1): strain J (JZ_058) was able to restrict phage L. In this strain LT2 sequence has replaced both *brxC* and *pglZ* of ER3625. The magnitude of restriction agreed with literature reports [43,44]: 100-fold reduction in plaque-forming ability when the test phage is unprotected (grown on non-modifying ER3625). We designed but did not create cassette-less strains with clean deletions of *brxB*, *ATPase*, *DUF3345* and *brxL*, which would be needed to test requirement for restriction. Intermediate strains M-Q, with *cat* cassette replacements, enable some conclusions (see below) but all have acquired resistance to phage L (Table 1).

**Fig 3:**
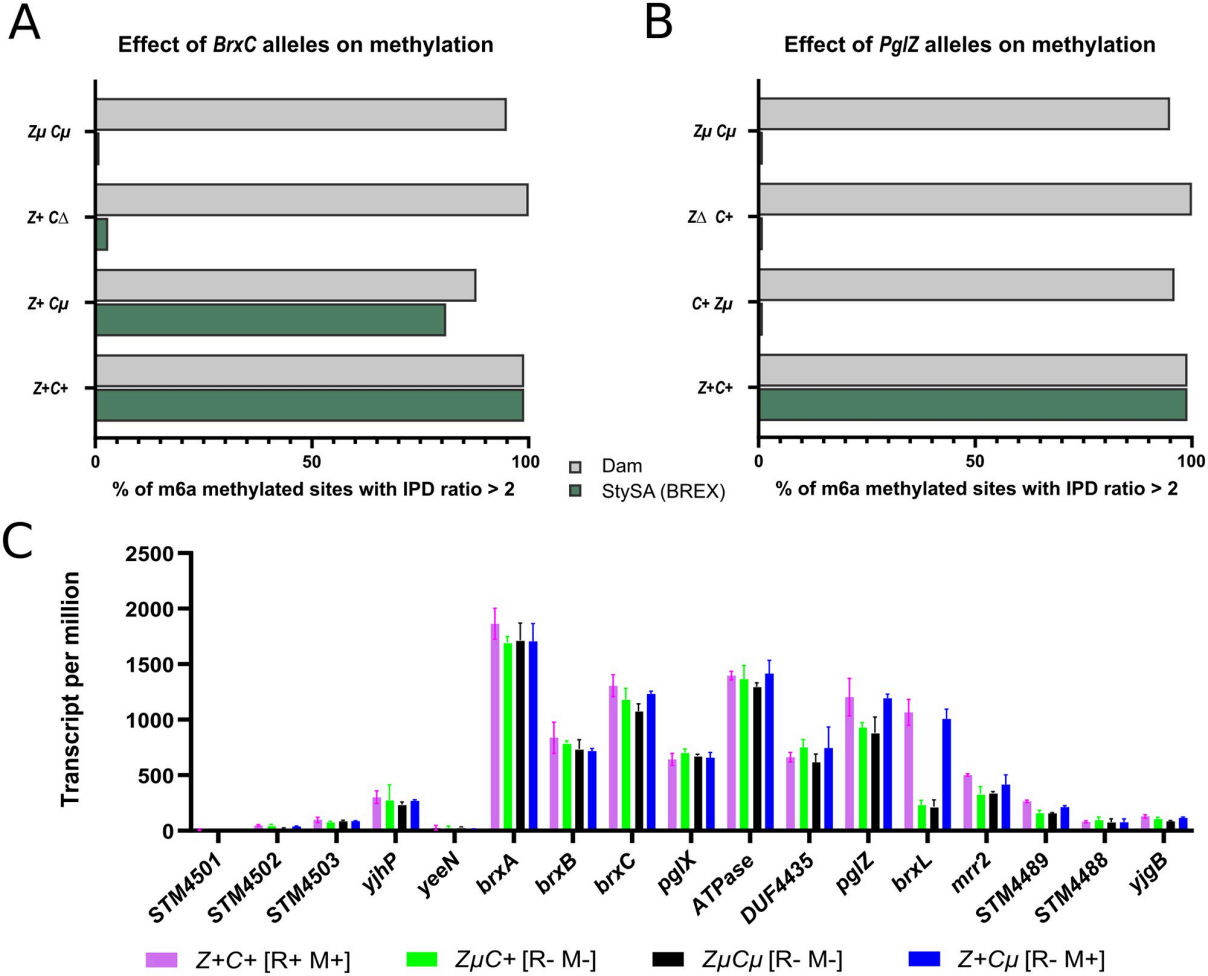
BrxC and PglZ alleles determine R and M phenotypes and BrxL transcript abundance. Panels A and B: effects of *brxC* (Panel A; strains A, D, B, J of Table 1) and *pglZ* (Panel B, strains A, E, C and J of Table 1) alleles on methylation of StySA sites (GATC**A**G green bars), with Dam sites (G**A**TC; gray bars) serving as a control. X-axis: fraction of sites methylated measured by delay of incorporation time (IPD) opposite A in the site relative to unmodified A. Y axis: genotype. Panel C: Transcription level across CDSs in the StySA neighborhood for four strains (J, C, A, B) with clustered mutations (μ) or WT (+) in *brxC* and *pglZ*. Strain restriction phenotypes are R^+^: 100-fold reduction in plaque formation with phage L; R^-^ no restriction activity. Vertical axis is transcripts assigned per million transcripts sequenced and is intrinsically normalized to feature size [101]. *pglZμ* is polar on *brxL*.

M phenotype was measured in genomic DNA by quantitating modification at the second A in GATC**A**G, using Pacific Biosciences IPD ratio measurements. Modification *in vivo* did not require WT BrxC: when *pglZ* is WT, mutated *brxCμ* allows modification (Fig 3A: *Z*^*+*^ *Cμ*). However, the N-terminus of BrxC is required for modification, since *ΔbrxC*::*cat* no longer modifies (Fig 3A: *Z*^*+*^ *CΔ*). In contrast, modification required PglZ to be intact: Zμ C^+^ does not modify (Fig 3B: *C+Zμ*).

#### *pglZμ* is polar on *brxL*

RNASeq measurements of transcription of the StySA locus (Fig 3C and S10 File) suggest that the alternative alleles (μ or +) do not affect transcription within the BREX cassette, with one exception: transcription of *brxL* is significantly increased when *pglZ* is WT (*Z*^*+*^*Cμ* or *Z*^*+*^*C*^*+*^) relative to *pglZμ* (*ZμCμ* and *ZμC*^*+*^). We infer that translation termination at the *pglZ* stop codon is polar on transcription of *brxL*. The phenomenon of translational polarity is well known though the mechanism is still under study (e.g., [45,46]); transcript quantity is decreased following a translation stop. However, intact BrxC is also needed for restriction: *Z*^*+*^*Cμ* does not restrict, so neither PglZ nor restoration of *brxL* transcription is sufficient when *brxCμ* is present.

#### Methylation activity does not depend on the level of *pglX* (MTase) transcription

We were unable to create a mutated *pglX* using our strategy. Instead, we asked whether changes in transcription due to engineering steps affect the degree of methylation. Using qPCR to measure *pglX* transcripts, we find that *pglX* transcript abundance does not correlate with M phenotype. M phenotype itself fell into three distinct categories (Table 1): StySA sites either were not modified at all or were completely modified, with only one genotype yielding an intermediate result. This intermediate modification property was replicated in three independently constructed strains of the same genotype (G,H, and I).

**Fig 4:**
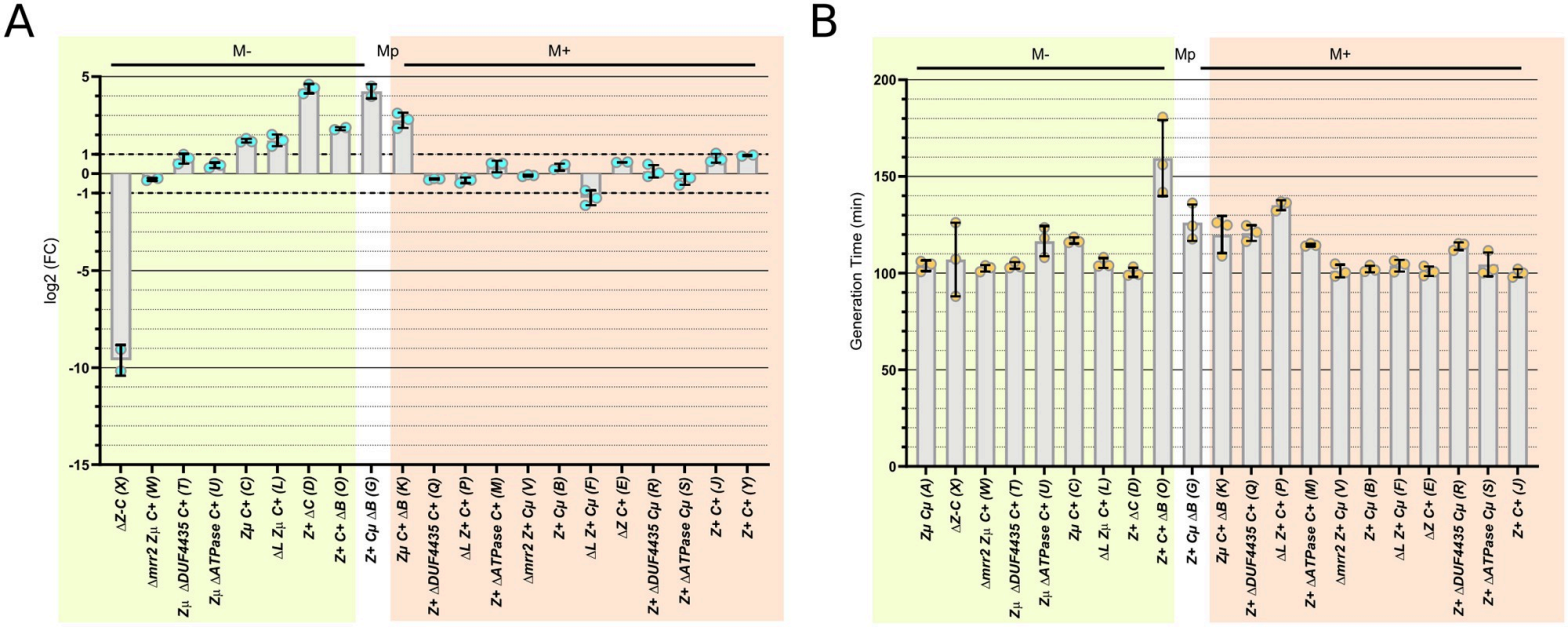
*pglX* transcript, M phenotype and growth rate responses to engineering. The X axis is common to Panels A and B, giving strain genotypes as in Table 1 (strains X and Y are not present in Panel B). Strains are grouped by M phenotype: green, M^-^ <6% of StySA sites are modified; white, M^p^ 46% modified; orange, M^+^ >83% modified. Panel A, qPCR measure of relative *pglX* transcript levels compared to the ancestor, strain A (log2fold change); a two-fold change is considered significant, indicated by the dotted lines at 1 and -1. Each blue dot shows relative transcript level for one biological replicate; bars display the mean for each strain. Panel B, generation time in minutes. Each orange dot represents a biological replicate; bars display the mean for the strain.

Modification categories did not reflect transcription level measured by qPCR (Fig 4A, M^+^, StySA modified; M^-^ StySA unmodified; M^p^, partial modification; S11 File). Significant differences in transcription are seen in some strains relative to ancestor ER3625, but the highest transcription is found in StySA M^-^ strains. A negative control is OD_127, a strain with a multigene deletion removing *pglZ-brxC* and including *pglX*.

#### Methylation activity is not affected by Δ*ATPase*::*cat*, Δ*DUF4435*::*cat* or *ΔbrxL*::*cat*

Removal of accessory genes *ATPase* or *DUF4435* do not affect methylation (Table 1 and Fig 4). Strains Q and R retain modification of their parents B or J M^+^ *pglZ*^*+*^ (*brxCμ* or *brxC*^*+*^) following introduction of *ΔDUF4435*::*cat*, as does strain S, *ΔATPase*::*cat* descendant of B; nor was modification restored to the M^-^ *pglZμ brxC*^*+*^ strain C when the accessory genes were removed in strains T and U. Similarly, removal of the N-terminal segment of *brxL* did not change M status (F, L and P strains agree with parents B, C and J). The contradictory properties of three *ΔbrxB*::*cat* strains are discussed further below.

#### Growth rate is affected by Δ::*cat* constructions

**Fig 5:**
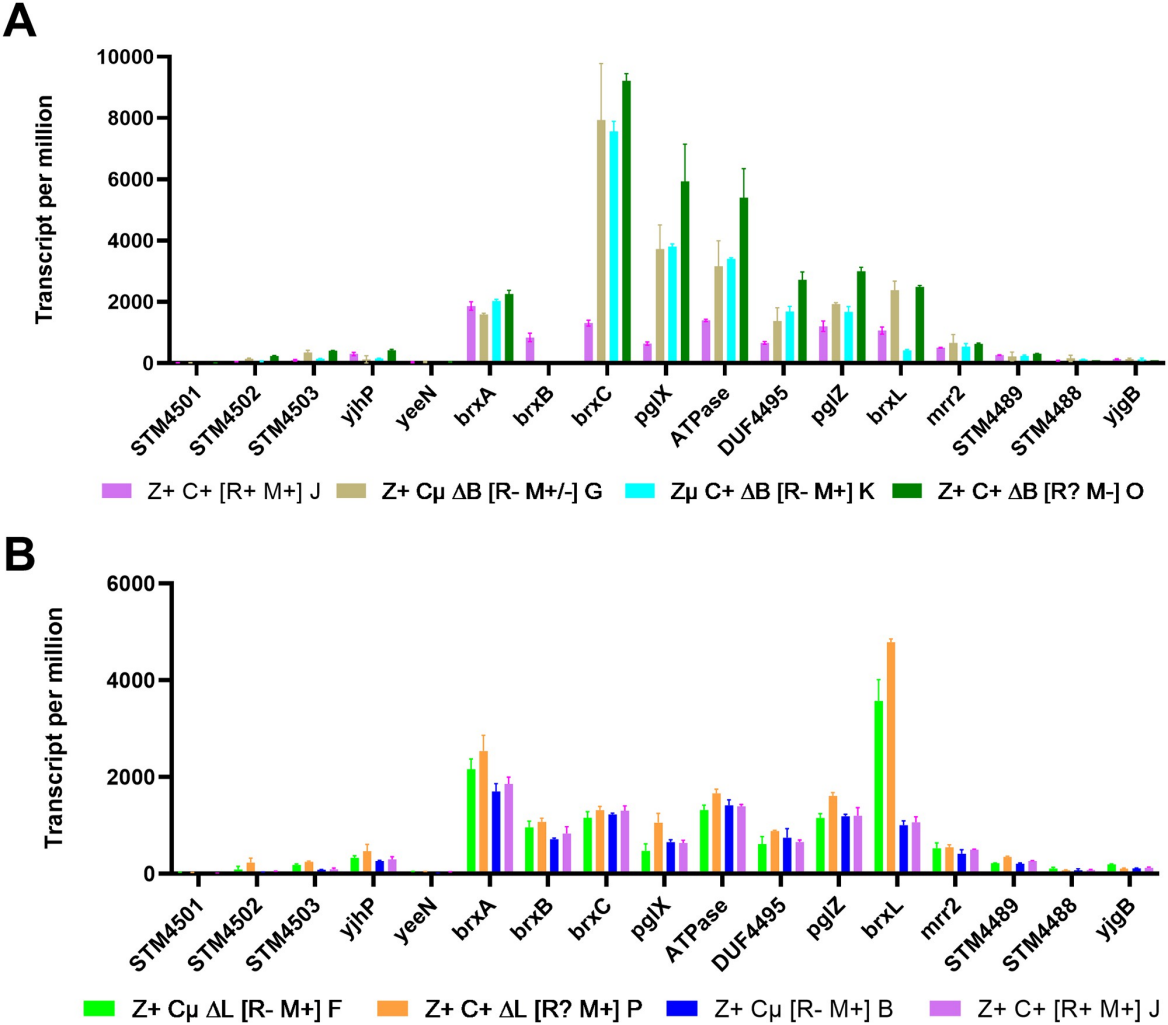
*Δ*brxC::cat and *Δ*brxL::cat each have a large effect on transcription of the StySA locus. The X axis displays CDS names in the StySA/BREX locus and its flanks. Ordinate is transcripts mapping to each CDS per million. Panel A: StySA/BREX WT and three combinations of *ΔB*::*cat* with *brxC* and *brxZ* alleles (Z+C+ [R+M+] strain J; Z+Cμ [R-M+/-] strain G; ZμC+ΔB [R-M+] strain K; Z+C+ [R?M-] strain O). Panel B: two *ΔL*::*cat* strains (F, P) paired with L^+^ ancestors (B, J). About 700 nt of the 3’ end of *brxL* is present in *ΔbrxL*::*cat*.

The strains have different colony phenotypes and growth rates. To better quantify this observation and accurately compare relative growth, we performed a liquid culture experiment (Fig 4B and S12 File). Most strains show growth rates like the ancestor ER3625. Interestingly, there is no correlation between methylation level and growth. However, the strains with *ΔbrxB*::*cat* share slower growth rate, regardless of the allelic state of *brxC* and *pglZ*. We attributed this shared property to the unbalanced transcription of the whole operon due to the *cat* cassette promotor, see below and Fig 5. Other slow growers are derivatives of WT strain J (P, Q and M) with *Δ*::*cat* insertions positioned to drive expression of *pglZ* and *brxL* (Q and M) or of the *brxL* fragment (P).

### Local and global inferences from transcriptome profiling

#### Unbalanced transcript abundance of BREX locus components with *ΔbrxB*::*cat*

The modification properties of strains with deletion/replacement of *brxB* are contradictory. In the genetic context of unmethylated strain C (*brxC*^*+*^ *pglZμ*), the *cat* replacement of *brxB* restores methylation in its derivative strain K (Table 1). In contrast, fully modified strain B (*brxCμ pglZ*^*+*^) becomes the partially modified strain G when Δ*brxB*::*cat* is introduced. This was reproduced in three independent constructions: strains G-I all have the same engineered genotype and similar intermediate modification levels. Meanwhile, completely WT strain J (*brxC*^*+*^ *pglZ*^*+*^) loses all StySA modification in strain O with Δ*brxB*::*cat*. In Discussion, we suggest that component ratios may account for this.

The "partially methylated" phenotype was reproducibly found in the genotype *brxCμ pglZ*^*+*^ *ΔbrxB*::*cat*. Three independent constructions (G-I, Table 1) with the same configuration all display the same modification pattern. Dam sites are methylated normally. Examining the distribution of modified StySA sites among these isogenic strains, we observed in each a patchy modification pattern. Multiple nearby StySA sites have very low modification while others comprise fully modified regions, rather than consistent half-modification (S5 File).

The RNAseq results for *ΔbrxB*::*cat* strains G, K and O also support effective action of the terminator between *brxC* and *pglX*: the transcript drops by half between these genes (Fig 5A and S10 File). This is consistent with the evidence for effective transcription termination at the end of *brxC* (see above "Transcription overview" and S8 File). Termination is not complete, allowing readthrough to *pglX*.

#### Overexpression of *’brxL* yields global effects without leakage across the insulating terminator

RNAseq results for *ΔbrxL*::*cat* strains provide strong evidence that the right-hand terminator insulates the flanking sequence from readthrough (Fig 5B and S10 File). Inside the locus, only the 707 nt remnant ’*brxL* gene has altered transcription; this is strongly increased relative to isogenic *brxL*^*+*^.

**Fig 6:**
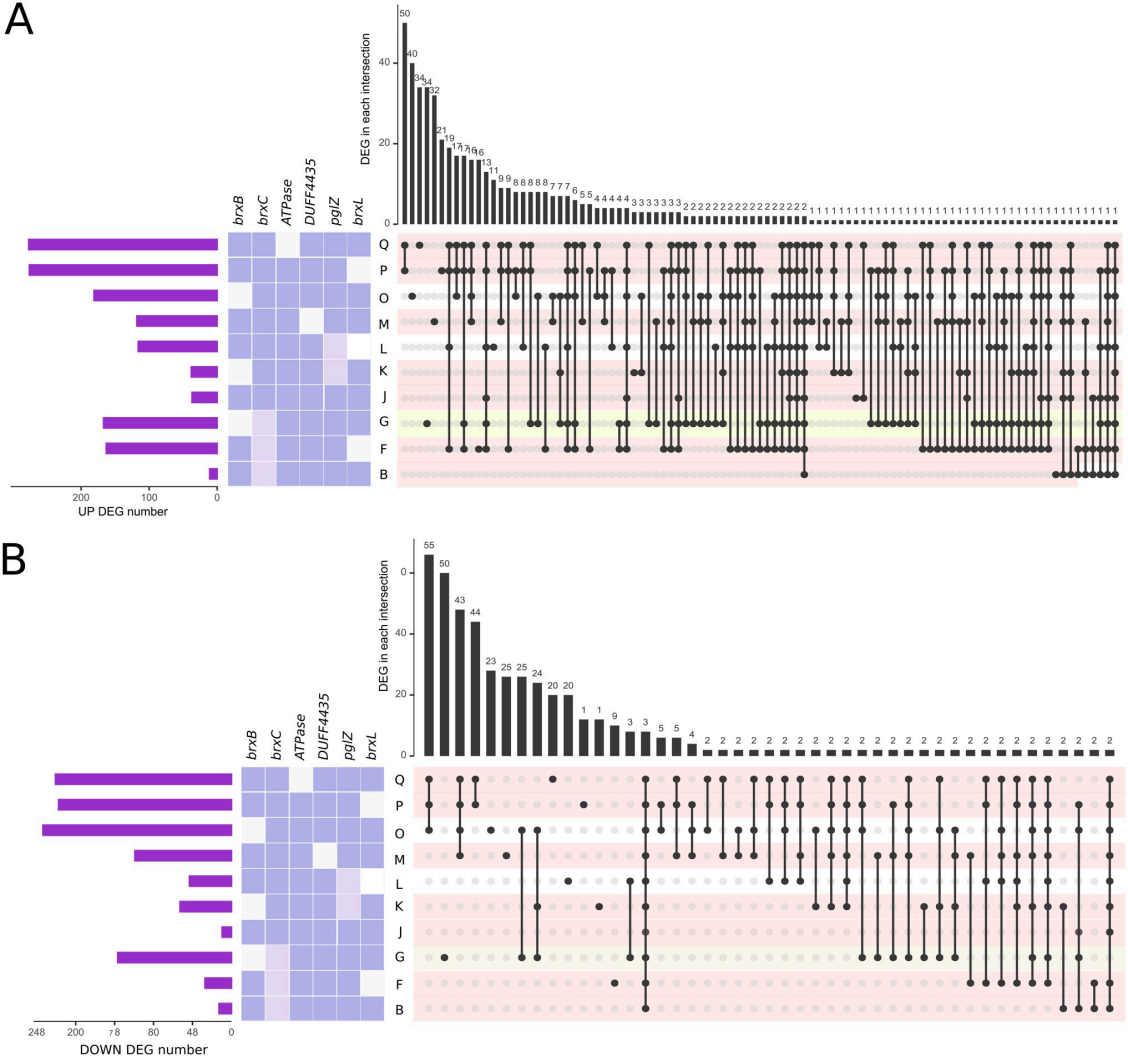
Global changes in transcription visualized with UpsetR. Differentially expressed genes (DEG) genes were evaluated for each CDS feature annotated in the ancestral sequence (ER3625) using RNASeq reads. Each row displays results for one strain. Changed transcription was reported as adjusted pvalue (see text). In the leftmost column, the purple bar chart shows the total number of DEG for the strain. In the middle, each the genotype is color coded: white, *cat* cassette replaced a segment of the CDS; dark purple, WT; light purple, μ allele inherited from ER3625. Strain code is from Table 1. On the right, sets of genes with changed transcription that are shared with other strains are represented by dots connected by lines. In each row, a black dot represents a set of genes in a particular strain. Between rows, lines connect that dot (set) to other strains with dots representing the same set. The histogram at the top indicates the number of genes in that set (intersecting group). Panel A: upregulated; Panel B: downregulated sets of genes. In each row, methylation phenotype is coded pink (M^+^), yellow (Mpartial) and white (M^-^).

There are other phenotypic effects of this disruption design. Growth rate is impaired for *Z*^*+*^ *C*^*+*^ *ΔL* (strain P) though not *Z*^*+*^ *Cμ ΔL* (strain F) or *Zμ C*^*+*^ *ΔL* (strain L) (Fig 4B); for all three strains, structural genes for two prophages are overexpressed, as well as numerous other genes outside the locus (Fig 6 and S4 and S9 Files).

#### Global inferences from transcriptome differential transcription profiling

The three engineered strains lacking *cat* cassettes are least globally affected in terms of gene transcription (Fig 6). The strain with unchanged R^-^M^-^ phenotype (strain C, *brxC*^*+*^ *pglZμ*) also has an unchanged global transcription pattern and does not appear in Fig 6). Strain B (*brxCμ pglZ*^*+*^) has recovered modification (M^+^); outside the locus, 11 and 8 genes show increased and decreased transcription respectively. Strain J (*brxC*^*+*^ *pglZ*^*+*^) has recovered both M^+^ and restriction (R^+^) with 37 changes up, 6 changes down. Of the 11 genes with increased transcription in M^+^ strain B, 2 are unique: a possible operon of genes JJB80_02810 (PLP-dependent aminotransferase) and JJB80_02815 (M15 family metallopeptidase). Of the 37 genes increased in R^+^M^+^ strain J, 1 is unique: JJB80_02375, a ribosomal protein associated with changes in frameshifting. It seems unlikely that these extra-locus changes are responsible for the M and R phenotypes.

A very striking result is that strains with *cat* cassettes (white boxes in the grid) have very large numbers of genes with significant changes in transcription, and the sets of genes with altered transcription are not widely shared (Fig 6). The chloramphenicol acetyltransferase enzyme (Cat) itself is not a good candidate cause. It is not a drug effect since no chloramphenicol was present. Cat might conceivably act on non-target metabolites, but if so all the *cat*-containing strains should have similar transcription impact. However, both number of genes affected, and the shared sets are quite different. For example, a small number of genes are affected in strains K and J; J is WT (*brxC*^*+*^ *pglZ*^*+*^) with no cassette, while K has *ΔbrxB*::*cat* combined with *pglZμ*. The three *ΔbrxL*::*cat* strains P, L and F have a much larger effect; in fact, the largest effect is strain P, which is otherwise WT.

Instead, it is likely that unbalanced transcription of elements of the StySA island mediate these drastic global effects. In most cases six, three or two downstream genes are overexpressed. Possible action on off-target substrates by helicase, methyltransferase, ATPase, and phosphatase activities is greatest for Δ*brxB*::*cat* (strains O, K and G) with over-transcription of *brxC*, *pglX*, *ATPase*, *DUF4435*, *pglZ* and *brxL*. For *ΔATPase*::*cat* (strain Q) downstream genes are *DUF4435*, *pglZ*, *brxL*. For *ΔDUF4435*::*cat* (strain M), only *pglZ* and *brxL* are overexpressed. In general flavor, the global changes in *ΔbrxB*::*cat* strains affect the three prophages in the strain and cell surface composition (S4 File). We did not see expression of SOS (DNA damage inducible) genes reported for *Salmonella enterica* serovar Typhimurium [47]. The patterns also do not resemble the *E*. *coli* response to the loss protective methylation in the presence of a restriction endonuclease [48].

Global changes in the *ΔbrxL*::*cat* strains P, L and F are more interesting, because the local effect of the *cat* insertion is limited: only the fragment of the *brxL* gene, ’*brxL*, is overexpressed (Fig 5B and S10 File). Genes lying outside the StySA cluster are insulated from transcription emanating from within the StySA cluster by a very strong terminator. The strong effects on global transcription (Fig 6 and S4 File) suggest that the *’brxL* transcript or a translation product act outside the locus.

We favor the model that this partial gene is translated into a stand-alone protein embodying the C-terminal domain (Lon_C, SSF54211: "Ribosomal_S5_D2-typ_fold"; see S6 File). The 5’ 1278 nt segment of the gene has been replaced with the *cat* cassette, but the 3’ 807 nt still carries an ORF. Translation from the original *brxL* N-terminus ends within the added *cat* cassette (118 nt *brxL* + 10 nt cassette). A transcript from the *cat* promoter could allow downstream translation restart following the *cat* CDS, at a CTG 20 nt into the *brxL* remnant (52 nt from the *cat* UGA). Such translation would yield a protein carrying the signature motifs from three domain annotation sources: GENE3d:3:30 Ribosomal_S5_D2-typ_fold_subgr; PFAM: Lon_C; Superfamily SSF54211 (Ribosomal_S5_D2-typ_fold). The candidate activities of the C-terminal domain include hydrolysis, phosphoryl transfer and folding, any of which could explain major cellular effects.

## Discussion

### BREX migration

#### StySA was recently acquired then embellished

Why is StySA/BREX DNA so similar to its counterpart *E*. *coli* HS BREX? The *brx/pgl* genes display 70–80% overall identity in Fig 1--considerably higher than the ~50% expected for *E*. *coli* and *S*. Typhimurium core gene orthologs [49,50]. Their ancestry is likely more recent than the divergence of the host taxa. Consistent with this, the two elements are found within variable islands, but at different genomic loci: in *S*. Typhimurium the island is at tRNA *leuX*, while in *E*. *coli*, a quite different island is at tRNA *thrW*. Distinct integrases of the P4 family are associated with these islands. In a study of the highly variable *leuX* genome islands of *Salmonella enterica*, Bishop and coworkers [37] used Southern blots, microarrays, and PCR to track residents at *leuX* in *S*. *enterica*, proposing a two-step acquisition of the BREX locus *per se* by *S*. *enterica*, followed by later acquisition of the ATPase-DUF4495 (STM4493-4) in serovar Typhimurium. Bishop et al also noted *in silico* similarity to database deposits of mobile elements from *E*. *coli* (AF550679), *Vibrio cholerae* (AY055428) and *Providencia rettgeri* (AY090559).

#### Transcription: Containment and opportunities for component regulation

Mobile elements frequently employ self-contained regulatory circuits that can make them substantially independent of a particular host [51]. These may take advantage of sRNA regulation, providing compact regulation [52]. Mapped TSS identified in our work provide opportunities for small RNAs within or between CDS on the coding strand or on the other strand (Fig 2).

Our map of transcription start sites and Rho-independent terminators is a start for understanding how this island is regulated. The variable transcript abundance across the locus shown will be product of initiation, termination, and degradation. Rho-dependent terminators are difficult to predict accurately but likely to be present and important [53]. RNA degradation is affected by the presence of protective structures, including annealed small RNAs [54].

Supporting the idea of a self-contained element, we see highly effective transcription termination adjacent to *brxL*: the very strong *cat* promoter drives high expression of the *brxL* 3’ remnant put gives no signal for the adjacent gene *mrr2* gene (Fig 5B).

A Rho-dependent terminator site or a stabilizing feature for *brxA* may lie between *brxA* and *brxB*. Without interference from a drug cassette, four of our strains display a drop in abundance there. This is followed by a rise for *brxC*. New transcript initiation could be then contributed by the TSS upstream of *brxC* (Fig 2). We presented evidence for action of the Rho-independent terminator following *brxC* (S8 File); hairpins that are part of the terminator can also function as stabilizers, potentially preserving *brxC*. *ATPase* may be transcribed from within *pglX*, followed by a drop for DUF4435 and a new TSS within the end of DUF4435 transcribing *pglZ-brxL*.

Translation into protein provides another possible layer of regulation, at which threshold effects are possible: translation of a transcript may be suppressed by a constitutive sRNA until overcome by increased transcription of the target [55,56]. Transcript stability or and/or processing can also affect abundance of encoded proteins. Small transcripts may also encode small proteins with their own regulatory activities [57].

### Toxic genetic states

The R^-^M^+^ phenotype originally reported for the lineage used in this work [36,44] was unstable, losing modification (M^+^) ability [58]. Here we observe a moderate growth defect with *brxCμZ*^*+*^ (R^-^M^+^ strain C) but not *brxC*^*+*^*Zμ* (R^-^M^-^ strain B). If BrxZ were toxic when not restrained by BrxC, selection for inactivating mutations might be observed.

The *ΔbrxA*::*cat* design was unsuccessful in three genetic contexts (*brxCμZ*^*+*^, *brxC*^*+*^*Zμ* and *brxC*^*+*^*Z*^*+*^). This might be expected if BrxB were a toxin and BrxA an antitoxin. Two other systems yielded conflicting results for *ΔbrxA*: deletion abolished both R and M with the *Acinetobacter* system [59]; in the *E*. *coli* system neither R or M depended on it [30].

We were also unable to recover a deletion of *pglX* in three allelic contexts (*brxCμZ*^*+*^, *brxC*^*+*^*Zμ* and *brxC*^*+*^*Z*^*+*^), even with the *brxC*^*+*^*Zμ* parent strain already lacking modification. Potentially, protein expression driven by *cat* transcription in the intermediate (ATPase, DUF4435, PglZ or PglZμ and/or Brx*L*) resulted in a toxic effect not found otherwise (Table 1 and Fig 4 and S1 File). We have not evaluated potential translation of a truncated PglX protein.

### Components of StySA methyltransferase

#### PglX as methyltransferase catalytic component and coordinator

We presume that PglX is required for StySA site-recognition and catalysis. DNA MTases transfer a methyl group from S-adenosyl methionine to a base in DNA. Cofactor binding and catalysis motifs associated with those activities are present in StySA PglX. MTases modify varied DNA sites, which are recognized by variable target recognition domains in the amino acid sequence [60,61]. The divergence in alignment between *E*. *coli* HS with Salmonella StySA-BREX within *pglX* (Fig 1) fits this picture.

Others have demonstrated PglX requirement for modification activity *in vivo*. Both distant and close PglX homologs have been shown to be required for modification. Distant examples are in high-GC (*Streptomyces coelicolor* [25]) and low-GC (*Bacillus cereus* [28] and *Lactobacillus casei* [32]) taxa, while closer relatives include *Acinetobacter* [59], *E*. *coli* HS [30] and the plasmid borne pEFER of *Escherichia fergusonii* ATCC35469 [17]. DNA modification is a protective function in the *E*. *coli* case, demonstrated using phage passage experiments and PacBio assessment of modification level.

In vitro confirmation of DNA methylation has been reported only for the original Pgl system in *S*. *coelicolor* [25]; PglX expression was carried out in *E*. *coli* in that case. For the Pgl system, modification results in sensitivity to restriction, not protection. No *in vitro* methylation experiments have been reported for BREX systems, in which *in vivo* methylation is protective.

Sensitivity to phage-encoded anti-restriction functions provides evidence that elements of BREX, particularly PglX, are likely to be related to Type I RM systems. Both StySA-BREX and *E*. *coli* HS BREX interact with phage anti-restriction activities previously thought to be specific for Type I enzymes. Both the DarAB activity of EcoP1 [35] and the Ocr activity of phage T7 [34] act against the StySA-BREX system.

Ocr, a DNA mimic [62,63], will defeat both R and M activities of the *E coli* HS BREX system [31]. Action is directed by interaction with BrxX (PglX): pull-down experiments with Strep-tagged Ocr successfully pulled down both BrxX (PglX) and the Type I MTase M.EcoKI in the same cells [31]. Tagged BrxZ (PglZ), BrxB, and BrxL did not interact with Ocr in this setup.

The antirestriction activity of phage P1 comprises proteins processed during phage morphogenesis, packaged into the virion, and delivered to the host during infection [64,65]. The injected DarB protein was noted to contain a bioinformatic signature of methyltransferase [65]. How exactly it acts against conserved RM systems with disparate recognition sequences has not been elucidated. Interference with integrity of the restricting assembly might play a role in disrupting both StySA-BREX and the Type I enzyme action.

Our contribution is to show that the degree of modification of the host DNA is not correlated with the level of transcription of *pglX*: the highest transcription occurred in strains that were unmodified. Among the possible explanations are aberrant translation (e.g. by titrating a regulatory sRNA) or titration of other protein components needed to assemble an active complex.

#### BrxC and PglZ as participants

Our data show that the N-terminal domain of BrxC and the complete PglZ protein are required for DNA modification, while the C-terminus of BrxC is also required for restriction (Table 1). These requirements are unlikely due to effects on transcription of the respective proteins, since modification and restriction are restored without notable effects on transcription within the locus, except for relief of polarity on *brxL* when *pglZ* is WT (Fig 3 panel C).

**BrxC**. We infer that the C-terminal stretch of BrxC that is altered in BrxCμ has DNA-interaction activity. This is based on detection of domain signature "SMC_prok_B" (TIGR02166) when searching InterProScan [66] in the Geneious implementation. Hits include an N-terminal ATPase region (P-loop NTPase, ATPase involved in DNA repair, P-loop containing nucleoside triphosphate hydrolase) and the C-terminal SMC (Structural Maintenance of Chromosomes) hit. In ER3625, the four mutations in *brxCμ* are clustered in the 3’ end of the gene. Presumably the variant amino acids result in loss of the SMC-domain recognition by the programs.

The SMC domain at the BrxC C-terminus could affect DNA conformation within a complex. For example, an ability to detect presence of multiple unmodified sites *in cis* is common for those RM systems with asymmetric sites (see, e.g., [67–70]). Such detection could act to license BrxL action to arrest phage development.

**PglZ** is a putative phosphatase, originally identified in the Pgl system. The Pgl system includes a protein kinase, PglW, thought to phosphorylate a component of the methyltransferase activity to prevent lethal self-modification. Such a modified protein would then provide the target of potential PglZ action. Confirming the relevance of the PglZ phosphatase signature, point mutations of the candidate catalytic aspartates could not be made in single copy [25]: so catalysis with that domain on some target was lethal. The model for action posits that unscheduled modification is lethal to the host, so posttranslational control is imposed by the kinase activity of PglW. PglW was shown to phosphorylate at least itself. A possible red herring is a potential nuclease activity in the PglW N-terminal NERD domain (a member of the PDDEXK clan [71]).

Shut-down of DNA methylation is not needed in BREX systems, which display the more-familiar RM paradigm of self-protection by modification [30] and do not have associated PglW homologs. Since no other kinase homologue is present in BREX systems, the potential phosphatase target is obscure. Considering the divergence of phenotypic endpoint as well as amino acid sequence, it may be that some other target is relevant, such as a nucleotide cofactor, or even some other phosphate-containing compound.

From our data, we conclude that the intact *pglZ* gene is required for both R and M activity *in vivo*, except in a single case discussed below.

#### Role of BrxB and component ratios in *ΔbrxB*::*cat*

We show that BrxB is also not required for methylation activity and is unlikely to participate in the active complex, though it could act as a chaperone or to support subcellular localization. The discussion here turns on strains in which *brxB* has been replaced with a drug cassette with a very powerful unregulated promoter, leading to excessive transcription of all genes in the locus (Fig 5A).

It is often observed that relative component abundance can affect the *in vivo* properties of DNA-active enzymes. Overexpression of proteins missing DNA-binding subdomains results in inhibition of McrBC restriction [72–74] and Tn5 transposase [75], a "titration effect". Simple overexpression of the WT protein is itself inhibitory for Mariner transposition. Enough protein can saturate available DNA target sites without demanding the synapsis needed for effective transposition [76–78]. With multicomponent transcription complexes, overproduction of an interaction domain can squelch activation [79–81]. This phenomenon has been used to survey interaction surfaces using overexpressed peptide fragments [82].

The *ΔbrxB*::*cat* allele results in contradictory effects on modification in the three genetic contexts (Table 1), which we attribute to effects on component ratios rather than its participation in the modification reaction.

In two other BREX systems, a clean deletion of *brxB* alone resulted in loss of modification [30,59]. In agreement with that result, replacement of *brxB* with *cat* in the WT context (M^+^ strain J) results in loss of modification in resulting M^-^ strain O.

In contrast, the M^-^ *brxC*^*+*^*pglZμ* strain C *gains* modification when *brxB* is replaced with *cat*, creating M^+^ strain K. This argues against a role for BrxB in the modifying complex itself. Extra BrxC combined with the PglZμ fragment and PglX may provide the needed component arrangement. As noted above, PglZ^+^ is required for modification in all other strains. We considered the possibility that massive transcript overproduction could result in translational readthrough allowing functional PglZ to be made in some amount from the *pglZμ* allele. However, strains S (*brxC*^*+*^*pglZμ ΔATPase*) and T (*brxC*^*+*^*pglZμ ΔDUF4435*) should yield even more transcription of *pglZμ*, but do not overcome the modification defect.

Finally, when *ΔbrxB*::*cat* is introduced into M^+^ *brxCμ pglZ*^*+*^ strain B, the resulting strains (G, H, I) display patchy intermediate methylation. This effect might be explained by a version of the "titration effect" seen with inhibition of McrBC assembly. There, the McrB_S_ protein carries the same NTP-binding domain as McrB_L_ but lacks the DNA-binding domain. Co-assembly into hexameric rings sequesters the cleavage component, McrC. Here, excess BrxCμ may interfere with but not abolish function of PglZ:PglX.

### StySA action and BrxL

The BrxL protein is critical to restriction action but not required for modification in other systems [30,59]. Our results agree that host modification does not require BrxL. Speculation on what BrxL targets and how it acts are constrained by several observations. First, the restricting complex must "count" unmodified sites, because more sites in the target lead to more severe restriction: such "counting" was observed for restriction of transformation by the *L*. *casei* system [32], and is also apparent with StySA. Phage L has 13 sites [83] and yields 100-fold restriction, while P22, with 3 sites, was so poorly restricted (~2 fold) that we did not collect EOP data systematically. Thus, the much or all of the unmodified target must be available for scanning inside the cell. Site recognition must occur in the presence of PglX. At least BrxC with its C terminus intact is involved in licensing BrxL action and might play a role in the scanning event.

#### A C-terminal BrxL fragment affects transcription globally

The C-terminal domain is of particular interest here, in view of the genome-wide transcription effects we observe with overexpression of the 3’ end of the gene. Translation of this would yield a C-terminal fragment with signatures of Lon protease C-terminus missing catalytic residues. We note that a similar Lon-related C-terminus is required for RadA/Sms to assist branch migration during DNA recombination in both Gram^+^ [84] and Gram^-^ [85,86] bacteria. Fork intervention delivered by the StySA holoenzyme only to multiply-unmodified DNAs is a tantalizing prospect.

The fragment alone has global effects. By inspection, we did not see induction of markers of DNA damage [47] and or of lethality upon loss of protective methylation in the presence of a restriction endonuclease [48]. Nevertheless, we did see enrichment for transcription of prophage genes, particularly Fels-1 and Gifsy-1 (S4 File). Regulation of transcription in these three prophages is tightly intertwined, mediated by anti-repressor interactions [87] and related to expression of pathogenicity functions [88], so the ’BrxL fragment may intervene at a single point that results in that enrichment.

## Materials and methods

### Terminology

- StySA: genes and phenotypes related to the cluster of genes that specify REBASE system *SenLT2II* of organism number 18099 and its lineal descendants [36]. This cluster is characterized genetically in this work, using ER3625 as experimental system. Most of the ER3625 genome is descended from LT2 with nitrosoguanidine mutagenesis; about 56 kb (0.1%) of the genome comprises two genome segments of 15 and 41 kb originating in *Salmonella enterica* serovar Abony SW803 [36].
- RM: restriction-modification system.
- R^+^, R^-^: restriction phenotype, measured by reduction in plaque formation of bacteriophage L when grown on a StySA M^-^ host [83].
- M^+^, M^-^, M^+/-^: genomic StySA sites (GATCAG) are fully (M+), not at all (M-) or partially (M+/-) methylated at the second A. Note that this sequence includes a Dam site (GATC); thus, the first A is also methylated.
- *gene*::*Δcat*: a gene with a portion deleted and replaced with the chloramphenicol resistance cassette of pKD3, including a strong promoter.

### Genome engineering

**Fig 7:**
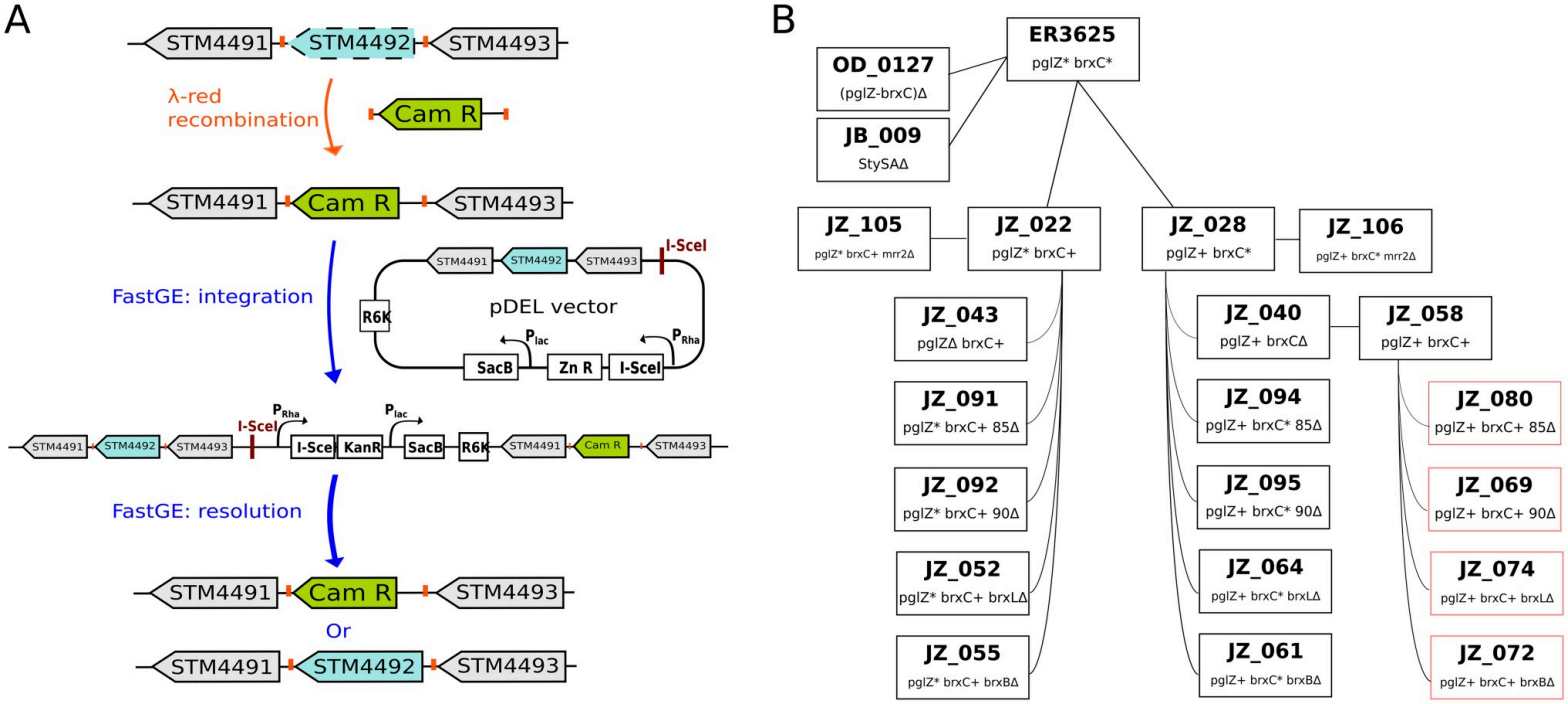
Engineering method and engineered strains. Panel A, Scarless engineering pipeline for replacement of mutated gene by WT. The lambda-Red recombination step is as in [89]. The integration and resolution steps are mediated by homologous recombination, with sucrose counterselection for excision [90]. Panel B, partial strain pedigree. Intermediate steps of panel A are omitted for clarity.

The Datsenko and Wanner method [89] was adapted and combined with the FAST-GE method [90] to engineer ER3625 descendants (Fig 7). During the second step of classic λ Red engineering, drug resistance cassettes would be removed by FLP/FRT recombination. For this, PCR-amplified *cat* cassette must be flanked by FRT (FLP Recombinase Target) sites such that the Flp recombinase can drive specific recombination between FRT sites. We found that resident FRT sites in ER3625 (*aroA*::*FRT*, *mrr*::*FRT*::*kan*) interfered with cassette addition to new sites, with the newly synthesized fragment recombining preferentially into the resident sites. Thus, in this work, the *cat* cassette was amplified and inserted without FRT sites.

#### Cassette replacement of a gene deletion or gene segment

A *cat* cassette flanked by 36–50 bp of homology to the target gene was PCR-amplified from pKD3 and used to electroporate an intermediate host carrying pKD46, a λ Red- expressing thermosensitive vector [89]. Colony PCR with flanking gene-specific primers validated the construction (S2 File).

#### Replacement of the *cat* cassette with WT sequence

The FAST-GE method was developed to rapidly engineer the genomes of both *E*. *coli* B and K12 strains with high efficiency by homologous recombination, with no residual extra sequence (“scar”) at the site of engineering. A single allele-exchange vector pDEL, was designed as compact as possible to maximize flexibility in application yield and fidelity. The pDEL vector contains several components involved in the promotion of recombination processes such as I-SceI endonuclease with its corresponding recognition site, and counterselection marker *sacB*. The unique double-strand break caused by I-SceI was shown to improve local recombination efficiency. Expression of *sacB* in the presence of sucrose is toxic to a variety of Gram-negative bacteria. To improve the overall efficiency of the protocol, *sacB* and gene coding for I-SceI endonuclease were placed under a *lac* and *rhaBAD* promoter respectively.

The original pDEL vector was constructed with a kanamycin resistance cassette as the primary selection marker. Since our host carries a kanamycin resistance cassette at the *mrr* locus, we designed two pDEL vectors with alternative antibiotic selection markers: zeocin and gentamycin (see sequences in S3 File). Both drug resistance cassettes were provided by Dr. Weigele. Zeocin is a bleomycin-like compound that kills both eukaryotic and prokaryotic cells by introducing lethal double-strand breaks in chromosomal DNA [91]. NEBuilder Hifi technology was used for construction of the pDEL-Zn (pOD003) and pDEL-Gm (pOD004) vectors (S1 File). Briefly, zeocin and gentamycin resistance cassettes were amplified by PCR with primers (oOD_070 and oOD_071) containing extensions homologous to the linearized pDEL vector without its kanamycin cassette (previously amplified with oOD_068 and oOD_069, S2 File), then assembled with the vector, transformed into *E*. *coli pir+* cells, selecting for the new drug resistance. Colony PCR with the set of primer oDO_072 and oDO_073 (S2 File) yields a product of 988 bp or 1.1 kb for pOD003 or pOD004 vector respectively. An amplicon of 48 bp is expected for the original pDEL vector (S3 and S2 Files).

This combination of methods was used successfully to engineer the strains characterized in this study for methylation and restriction activity (Table 1 and S1 File). Cappable-seq data (Fig 2) were used in the strain design to limit as much as possible the disruption of TSS and transcription terminator structures (S1 Fig.).

#### Linear method for locus replacement

This method consists of amplifying a cassette and flanking homology regions to replace a genome segment with the cassette. Here we tested several lengths of homology regions (3, 5, 9 and 12 kb). 3 kb worked. First, we amplified the Zn cassette from pJBJZ_006 with oJB_018 and oJB_017 and the genomic flanking regions with dedicated primers (oJB_009/oJB_006 and oJB_007/oJB_010). These fragments were purified with NEB T1030 kit and ligated together using the NEB E2621 NEBuilder Hifi DNA assembly with a ratio 1:1:1 (HR1: cassette: HR2) with a 30 min incubation. The assembly was then amplified using NEB M0493 Q5 High-Fidelity DNA Polymerase with an internal pair of primers (oJB_005/oJB_008) and repurified with NEB T1030 kit. Transformation was performed with 220 ng of the amplicons with Zn selection. Clones were verified by colony PCR.

### Phenotype tests

#### Growth media

Bacteria were grown in RB (10g soy peptone, 5g yeast extract, 5g NaCl per liter) or RBStrepKan (RB with Streptomycin (100 μg/mL) and Kanamycin (40 μg/mL) unless otherwise indicated.

#### Phage restriction tests

Bacteriophage L, with 13 sites for StySA [83] was used to test for StySA restriction, following the practice of the Colson and Ryu laboratories [44,92]. Bacterial strains were grown in RB with antibiotic overnight at 37°C with agitation. The cultures were subcultured in new RB media without antibiotic and grown until exponential phase at 37°C.

Two-layer agar plates were used for the spot titers. The bottom layer is 1.5% agar (per liter: 15g of Bacto Agar BD Biosciences #214030, 10g of Bacto Tryptone BD Biosciences #211699, 5g of Bacto Yeast extract BD Biosciences #212720 and 5g of NaCl) and the top layer is an agar 0.7% (same recipe as the bottom layer but only 7 g of Bacto agar). Bacterial cultures were mixed with the top agar layer (56C) and poured on the bottom layer. The bacteriophage stocks (PH_JZ003 and PH_JZ006, see details in S1 File) were diluted from 10^−1^ to 10^−8^; 5 μl of each dilution was spotted on the plates; incubated at room temperature until dry, and incubated 18h at 37°C. Strains ER3625 and ER3649 were negative and positive controls. Plaques were counted on spots where they were well isolated.

#### Growth rate analysis

Growth rates were estimated using optical density (OD). ODs were measured in 96 well plates with a plate reader (Molecular Devices, SpectraMax ABS Plus) with two technical replicates of each of three biological replicates. A single colony was inoculated in 1 ml RBStrepKan in a deep well plate, then incubated overnight at 37°C with shaking 200 rpm. A 96 well plate (Greiner Bio-One ref: 655892) was prepared with 200μl RBStrepKan per well and inoculated with 2μl of the overnight culture. The growth was monitored every 15 minutes for 15 hours, between each measure, the plate was shaken.

Growth rates were calculated from the raw data using a rolling regression from: https://padpadpadpad.github.io/post/calculating-microbial-growth-rates-from-od-using-rolling-regression/.https://padpadpadpad.github.io/post/calculating-microbial-growth-rates-from-od-using-rolling-regression/. A one-way ANOVA for normally distributed samples of non-equal variance was performed on the data to determine statistical significance of growth differences.

### Nucleic acid methods

#### Genomic DNA (gDNA) extraction, sequencing

Each strain was growth in RB with the appropriate antibiotics (see "Growth media" above) overnight at 37°C with 250 rpm agitation.

gDNA was extracted with the Monarch Genomic DNA purification kit (New England Biolabs; Ipswich, MA, USA) from 1ml of culture.

Libraries from these genomic DNAs were sequenced using the PacBio RSII or Sequel I sequencing platform. Briefly for RSII, SMRTbell libraries were constructed from genomic DNA samples sheared to between 10 and 20 kb using the G-tubes protocol (Covaris; Woburn, MA, USA), end repaired, and ligated to PacBio hairpin adapters. Incompletely formed SMRTbell templates and linear DNAs were digested with a combination of Exonuclease III and Exonuclease VII (New England Biolabs; Ipswich, MA, USA). The SMRTbell library was prepared according to PacBio sample preparation protocol sequenced with C4-P6 chemistry with a 300 min collection time.

For Sequel I libraries, SMRTbell libraries were constructed from genomic DNA samples following the PacBio protocol for Sequel using the kit 100-938-900. DNA qualification and quantification were performed using the Qubit fluorimeter (Invitrogen, Eugene, OR) and 2100 Bioanalyzer (Agilent Technology, Santa Clara, CA). The libraries were prepared for binding following the PacBio guidelines generated by SMRT Link and run on a Sequel I machine.

#### RNA extraction

For preculture three isolated colonies were grown in a 1ml RBStrepKan or RBStrepKan with Chloramphenicol (30 μg/mL) overnight at 37°C in a deep well with breathable cover tape. The cultures were subcultured the next day in 25ml RBStrepKan (no chloramphenicol) at 37°C with 250 rpm agitation. Cells were harvested when OD 600 nm reached ~0.3 by centrifugation 10 min at 4°C. Pellets were resuspended in 100μl of cold 0.1X PBS.

RNA was extracted using the Qiagen RNA extraction kit following the classical protocol for bacterial RNA. The eluted RNA was then treated with DNAse I (NEB) and then cleaned and concentrated using a classical phenol-chloroform RNA extraction. RNA was stored at -80°C.

#### TSS determination using Cappable-seq method

The TSS Cappable-seq libraries were prepared following the recommendation of the protocol from reference [40] starting from 2μg of RNA in duplicates and controls. The libraries were run with a MiSeq with 1 X 75bp insert size using V3 Illumina platform.

The analysis was run in command line from the raw data using the script available at https://github.com/Ettwiller/TSS/.

Quantitation of *brxX* transcription employed qPCR. Primers used are listed in S2 File. The Lunascript RT Supermix kit E3010 was used for random conversion of 500 ng of extracted RNA to cDNA per samples (three biological replicates per strain) following the kits guidelines. The no-RT reactions were run on the same plate with the same RNAs. qPCR was then run with 2 primer sets, *yceB* (oJZ_116 and oJZ_117) and *brxX* (oJZ_150 and oJZ_151) using the Luna Universal qPCR Master Mix Protocol (M3003) with 1μl of cDNA per well. For each primer pair a standard curve was run on the same plate as the sample with 1, 0.1, 0.01, 0.001 and 0.0001ng and water only. All combinations were run in duplicate on the same plate. The plates were sealed and centrifuged for 30s and then run 39 cycles on the same CFX96 Touch Bio-Rad machine following the Luna kit recommended cycle steps.

### Sequence verification and methylation analysis

The BREX engineered locus sequence was verified in two ways. First, PCR reactions (primers oJZ_241/242, S2 File) with LongAmp polymerase (New England Biolabs; Ipswich, MA, USA), purified and sequenced (Sanger). Second, PacBio sequencing reads from the methylation analysis was aligned with the *in silico* design of the engineered locus.

DNA motifs and degree of modification were generated using InterPulse Duration (IPD) Ratios analyzed with RS_Modification_and_Motif_Analysis from Pacific Biosciences as in [93,94]. PBMotStat, a component of REBASE TOOLS [95] was used to calculate the % of methylated sites with IPD > 2 for specific sites.

For the partially methylated strains, a.gff file of the methylation level of each base of the genome was downloaded from SMRT Link, filtered to keep only significantly methylated sites, and uploaded in Geneious Prime on the reference genome (S5 File).

### Bioinformatic methods

#### Annotation of predicted functional domains and transcription signals

Predicted functional protein domains were annotated using Genbank-assigned protein IDs listed in S1 Table for LT2 and for ER3625. The NCBI protein IDs are automatically annotated with "regions" that correspond to Conserved Domain Database [96] concise predictions. Additional automated domain annotations were generated and visualized by submission to InterPro using the Geneious implementation of InterProScan as described in S6 File. Manual search of the Conserved Domain Database with "Full results" instead of "Concise results" also elicits annotations compatible with InterPro.

Potential transcription start sites were documented experimentally as described below (CappableSeq). Rho-independent transcription terminators were predicted using TransTerm HP algorithm version 2.09 ([97,98]), then curated manually.

#### Global and local transcriptome analysis by RNAseq

RNAseq libraries were prepared with the "protocol for library preparation of Intact RNA using NEBNext rRNA depletion kit (Bacteria) (NEB#E7850, NEB#78860) and NEBNext Ultra II directional RNA library prep kit for Illumina (NEB#E7760, NEB#E7765)" from 250ng of sample. Libraries were barcoded with dual index from NEB Multiplex Oligos for Illumina (96 Unique Dual Index Primer Pairs set 4, E6446S). The sequencing of the pooled libraries was performed on a NextSeq apparatus.

The raw reads were analyzed with Galaxy (Version 0.4.3.1) for QC, mapping, and feature assignment (workflow of S2 Fig). After a first QC step to assess the quality of the reads, they were trimmed using TrimGalore (Version 0.4.3) with parameters: -q 20 -s 3 -e 0.1 –length 20 and then a second QC steps was performed to verify the read quality after trimming. The trimmed reads were mapped with bowtie2 (with default parameters except–fr for upstream/downstream mate orientations) to the ER3625 genome (NCBI CP067091-CP067092 and [83,99]. The alignment was then used to count the number of fragments mapped to CDS and rRNA features using in parallel Featurecounts [99] (parameters: -s stranded (reverse)) and htseq (Version 0.9.1) with parameters:--mode Union–stranded reverse–minaqual 10 --idattr locus_tag–nonunique no. Results are displayed in Figs 2, 3 and 5 as TPM, which is intrinsically normalized to gene length [100,101]).

The differential analysis was performed using the DESeq2 R package [102] in command line. The parameters were set as: lfcShrink = apeglm, padj < 0.05 and log2FoldChange > |1.5|. UpsetR was used for visualization (Fig 6) [103]. Intersect (https://bedtools.readthedocs.io/en/latest/content/tools/intersect.html) [104] was used to generate lists of features common to chosen strain sets used in S4 File.

## Supporting information

S1 Table BREX homolog sources and labels. Locus_ID and PID for BREX loci.
(DOCX)

S1 Fig Deleted StySA-BREX segments. Segments replaced with *cat* related to TSS and TTS; the document includes a legend.
(DOCX)

S2 Fig Galaxy pipeline. RNAseq analysis workflow, includes a legend with link to Galaxy public web site.
(DOCX)

S1 File Plasmid strain phage. Excel file of Strain list with genotype and construction steps; plasmids with description and availability; phage with source and identity and RM phenotype of last host.
(XLSX)

S2 File Primers. Oligonucleotide sequences used for plasmid construction, strain engineering, validation of sequences and qPCR.
(XLSX)

S3 File Annotated plasmid cassettes. Component sequences annotated with locations of primers, coding sequences, promoters, terminators, replicons, and protein binding sites.
(DOCX)

S4 File RNAseq genome hits. Over- and under-transcribed gene sets for strain groups of interest; Genbank CDS list for reference, annotated with some genome island and prophage regions. Protocol for annotation transfer is in S9 File.
(XLSX)

S5 File Partial methylation patterns. This file combines a reference sequence and gff file for modification of independently constructed isogenic strains JZ_055, JZ_056, JZ_057.
(ZIP)

S6 File Potential BrxL activities. This includes three figures, an annotation protocol, and extended discussion of activities of other proteins with domain hits like that of the BrxL C-terminal domain.
(DOCX)

S7 File SRA Deposits. Tables of NCBI accessions with strain, NEB strain ID if applicable, Biosample Name (NCBI-assigned) and analysis type (RSII, Sequel, Cappable-seq, NextSeq RNAseq).
(XLSX)

S8 File Co-transcription and Transcript Termination Sites. Four figures: Sample isolation strategy and transcripts probed for *brxA*, *ATPase*, and *DUF4435* TSS visualized with IGV sequence read alignment screenshots at different magnifications.
(DOCX)

S9 File Protocol Annotation transfer to RNASeq files. (DOCX)

S10 File TPM data. Numerical data for Figs 2, 3C and 5.
(XLSX)

S11 File brxX qPCR data. Numerical data for Fig 4A.
(XLSX)

S12 File Growth data. Numerical data for Fig 4B.
(XLSX)
