# Supplementary material for: Reassembling a cannon in the DNA defense arsenal: Genetics of StySA, a BREX phage exclusion system in Salmonella lab strains
Source: PLoS Genet. 2022 Apr 4;18(4):e1009943. doi: 10.1371/journal.pgen.1009943 (PMC9009780; doi:10.1371/journal.pgen.1009943)
Supplement: S1 Table — Locus_ID and PID for BREX loci. (DOCX) [file pgen.1009943.s001.docx]

**S1_Table. BREX homolog sources and labels.**

| **Orth-olog** | **BrxA** | **BrxB** | **BrxC** | **PglX** | **ATPase** | **DUF4435** | **PglZ** | **BrxL** |
| --- | --- | --- | --- | --- | --- | --- | --- | --- |
| **Gene name in reference^a^** | *brxA* | *brxB* | *brxC* | *brxX* |  |  | *brxZ* | *brxL* |
| **Fig 1A label** | *brxA* | *brxB* | *brxC* | *pglX* |  |  | *pglZ* | *brxL* |
| ***E. coli* HS^a^ Locus_ID ECHD suffix** | RS01700 | RS01700 | RS01710 | RS01715 |  |  | RS01720 | RS01725 |
| **LT2^c^ Locus_ID STM suffix** | 4498 | 4497 | 4496 | 4495 | 4494 | 4493 | 4492 | 4491 |
| **Protein ID LT2 NP_suffix** | 463357.1 | 463356.1 | 463355.1 | 463354.1 | 463353.1 | 463352.1 | 463351.1 | 463350.1 |
| **Fig 1B label** |  |  | LT2-BrxC |  |  |  |  |  |
| **Fig 1C label** |  |  |  |  |  |  | LT2-PglZ |  |
| **Fig 2 label** | *brxA* | *brxB* | *brxC* | *pglX* | *ATPase* | *DUF4435* | *pglZ* | *brxL* |
| **ER3625 Locus_ID JJB80 suffix** | 22610 | 22605 | 22600 | 22595 | 22590 | 226585 | 22580 | 22570 |
| **Fig 3A label** | *brxA* | *brxB* | *brxCµ* | *pglX* | *ATPase* | *DUF4435* | *pglZ* | *brxL* |
| **Protein ID ER3625^d^** | QTQ02087.1 | QTP99676.1 | QTP99675.1 | QTP99674.1 | QTP99673.1 | QTP99672.1 | not assigned | QTP99671.1 |
| **Fig 1B label** |  |  | ER3625 BrxCµ |  |  |  |  |  |
| **Fig 1C label** |  |  |  |  |  |  | ER3625 PglZ' |  |

^a^[1]

^b^*Escherichia coli* HS - NC_009800.1 (340898 -> 354284); genome reported in [2]

^c^*Salmonella enterica subsp enterica* sv Typhimurium str. LT2 NC_003197.2 (4736695 -> 4751857); genome reported in [3]

*^d^Salmonella enterica subsp enterica sv Typhimurium* str ER3625 - CP067091 (4667817 -> 4694979); genome reported in [4]

REFERENCES

1. Gordeeva J, Morozova N, Sierro N, Isaev A, Sinkunas T, Tsvetkova K, et al. BREX system of Escherichia coli distinguishes self from non-self by methylation of a specific DNA site. Nucleic Acids Res. 2019;47(1):253-65. Epub 2018/11/13. doi: 10.1093/nar/gky1125. PubMed PMID: 30418590; PubMed Central PMCID: PMCPMC6326788.

2. Rasko DA, Rosovitz MJ, Myers GS, Mongodin EF, Fricke WF, Gajer P, et al. The pangenome structure of Escherichia coli: comparative genomic analysis of E. coli commensal and pathogenic isolates. J Bacteriol. 2008;190(20):6881-93. Epub 2008/08/05. doi: 10.1128/JB.00619-08. PubMed PMID: 18676672; PubMed Central PMCID: PMCPMC2566221.

3. McClelland M, Sanderson KE, Spieth J, Clifton SW, Latreille P, Courtney L, et al. Complete genome sequence of *Salmonella enterica* serovar Typhimurium LT2. Nature. 2001;413(6858):852-6. Epub 2001/10/26. doi: 10.1038/35101614. PubMed PMID: 11677609.

4. Zaworski J, Dagva O, Kingston AW, Fomenkov A, Morgan RD, Bossi L, et al. Genome archaeology of two laboratory Salmonella enterica enterica sv Typhimurium. G3 (Bethesda). 2021;11(9):jkab226-. Epub 2021/09/21. doi: 10.1093/g3journal/jkab226. PubMed PMID: 34544129; PubMed Central PMCID: PMCPMC8496262.
