# Supplementary material for: Reassembling a cannon in the DNA defense arsenal: Genetics of StySA, a BREX phage exclusion system in Salmonella lab strains
Source: PLoS Genet. 2022 Apr 4;18(4):e1009943. doi: 10.1371/journal.pgen.1009943 (PMC9009780; doi:10.1371/journal.pgen.1009943)
Supplement: S1 Fig — Segments replaced with cat related to TSS and TTS; the document includes a legend. (DOCX) [file pgen.1009943.s002.docx]

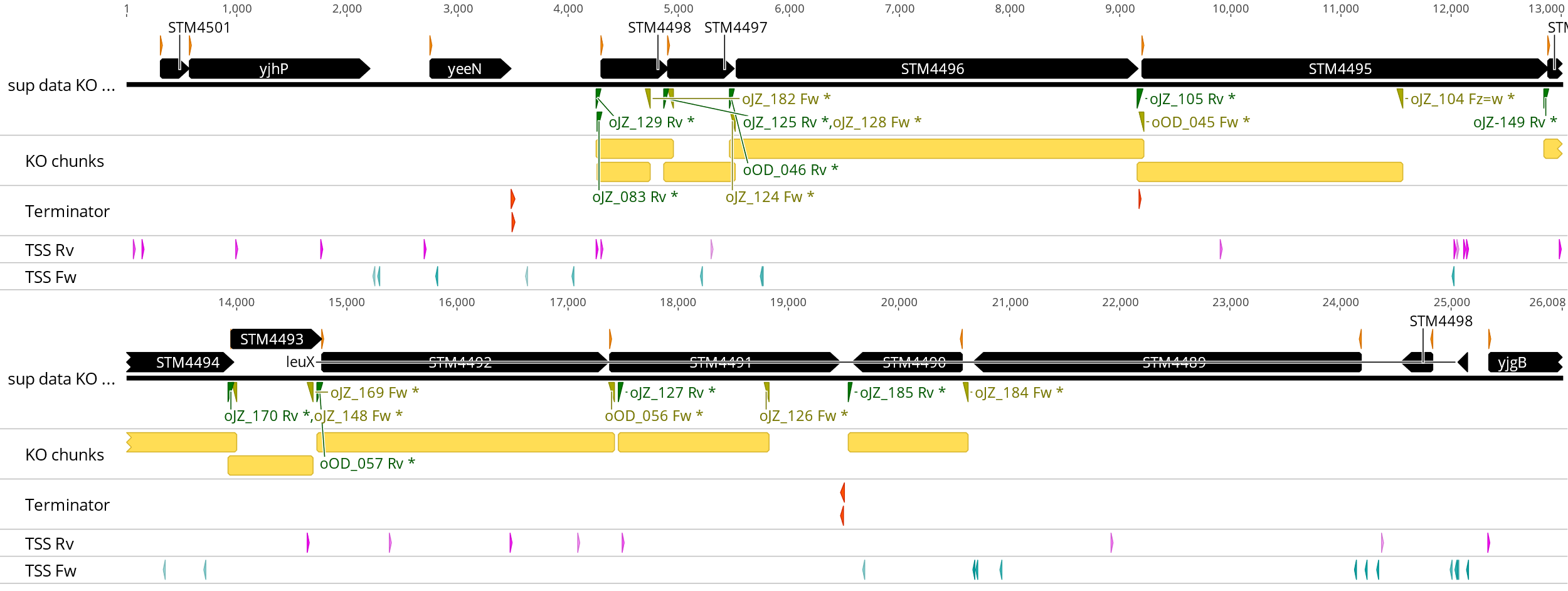


S1 Fig. Segments missing in *∆::cat* constructions related to TSS and TTS.

Top row: nucleotide coordinate of the sequence extraction used in the design process (from LT2 sequence NC_003197.2). Red arrows: annotated ribosome binding sites from NCBI; black arrowheads: CDS with LT2 locus_ID; green wedges: forward (dark) and reverse (light) primers used in constructions (see S3 File). Yellow blocks (KO chunks) correspond to the DNA segments removed and replaced with *cat* cassette in strains with *∆::cat* in the corresponding genotype. Terminator row: red arrows are at the positions of predicted rho-independent terminators. Two of the three locations are predicted to act bidirectionally. TSS Rv and TSS Fw: experimentally determined TSS from LT2 (our isolate STK013) in the Cappable-seq experiment of Fig 2. Fw and Rv are forward and reverse relative to the NCBI genome coordinate, not relative to the CDS
