# Supplementary material for: Reassembling a cannon in the DNA defense arsenal: Genetics of StySA, a BREX phage exclusion system in Salmonella lab strains
Source: PLoS Genet. 2022 Apr 4;18(4):e1009943. doi: 10.1371/journal.pgen.1009943 (PMC9009780; doi:10.1371/journal.pgen.1009943)
Supplement: S2 Fig — RNAseq analysis workflow, includes a legend with link to Galaxy public web site. (DOCX) [file pgen.1009943.s003.docx]

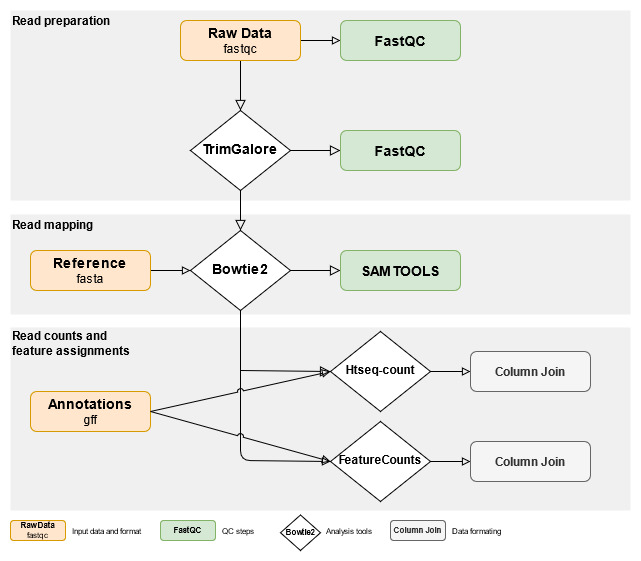


S2 Fig. Galaxy analysis pipeline used to generate fragment counts per CDS from the raw reads. The pipeline can be downloaded from the Galaxy public website: https://usegalaxy.org/u/j_zaworski/w/rnaseqpairedendtrimmapcountsmergejz
