# Supplementary material for: Reassembling a cannon in the DNA defense arsenal: Genetics of StySA, a BREX phage exclusion system in Salmonella lab strains
Source: PLoS Genet. 2022 Apr 4;18(4):e1009943. doi: 10.1371/journal.pgen.1009943 (PMC9009780; doi:10.1371/journal.pgen.1009943)
Supplement: S3 File — Component sequences annotated with locations of primers, coding sequences, promoters, terminators, replicons, and protein binding sites. (DOCX) [file pgen.1009943.s006.docx]

# Chloramphenicol cassette

**>Primer**

|

**taaatggcgcgccttacgccccgccctgccactcatcgcagtactgttgtaattcattaagcattctgccgacatggaagccatcacaaacggcatgatg** < 100

10 20 30 40 50 60 70 80 90

**aacctgaatcgccagcggcatcagcaccttgtcgccttgcgtataatatttgcccatggtgaaaacgggggcgaagaagttgtccatattggccacgttt** < 200

110 120 130 140 150 160 170 180 190

**aaatcaaaactggtgaaactcacccagggattggctgagacgaaaaacatattctcaataaaccctttagggaaataggccaggttttcaccgtaacacg** < 300

210 220 230 240 250 260 270 280 290

**<CmR**

|

**ccacatcttgcgaatatatgtgtagaaactgccggaaatcgtcgtggtattcactccagagcgatgaaaacgtttcagtttgctcatggaaaacggtgta** < 400

310 320 330 340 350 360 370 380 390

**acaagggtgaacactatcccatatcaccagctcaccgtctttcattgccatacgtaattccggatgagcattcatcaggcgggcaagaatgtgaataaag** < 500

410 420 430 440 450 460 470 480 490

**gccggataaaacttgtgcttatttttctttacggtctttaaaaaggccgtaatatccagctgaacggtctggttataggtacattgagcaactgactgaa** < 600

510 520 530 540 550 560 570 580 590

**atgcctcaaaatgttctttacgatgccattgggatatatcaacggtggtatatccagtgatttttttctccattttagcttccttagctcctgaaaatct** < 700

610 620 630 640 650 660 670 680 690

**<cat promoter**

|

**cgacaactcaaaaaatacgcccggtagtgatcttatttcattatggtgaaagttggaacctcttacgtgccgatca**acgtctcattttcgccaaaagttg < 800

710 720 730 740 750 760 770 780 790

**<Primer**

|

gcccagggcttcccggtatcaacagggacaccaggatttatttattctgcgaagtgat**cttccgtcacaggtagg** < 875

810 820 830 840 850 860 870

Features :

Primer : [1 : 17]

CmR : [673 : 14]

cat promoter : [776 : 674]

Primer : [875 : 859]

# Zeocin Cassette

**>UL1**

|

**caccaaacaccccccaaaacc**aagccactggagcacctcaaaaacaccatcatacactaaatcagtaagttggcagcaccacccgacgcactttgcgccg < 100

10 20 30 40 50 60 70 80 90

aataaatacctgtgacggaagatcacttcgcagaataaataaatcctggtgtccctgttgataccgggaagccctgggccaacttttggcgaaaatgaga < 200

110 120 130 140 150 160 170 180 190

**>promoter**

|

cgttgatcggcac**gtaagaggttccaactttcaccataat**gaaataagatcactaccgggcgtattttttgagttatcgagattttcaggagctaaggaa < 300

210 220 230 240 250 260 270 280 290

gctaaa**atggccaagttgaccagtgccgttccggtgctcaccgcgcgcgacgtcgtcggagcggtcgagttctggaccgaccggctcgggttctcccggg** < 400

310 320 330 340 350 360 370 380 390

**>Zeocin resistance CDS**

|

**acttcgtggaggacgacttcgccggtgtggtccgggacgacgtgaccctgttcatcagcgcggtccaggaccaggcggtgccggacaacaccctggcctg** < 500

410 420 430 440 450 460 470 480 490

**ggtgtgggtgcgcggcctggacgagctgtacgccgagtggtcggaggtcgtgtccacggacttccgggacgcctccgggccggccatgaccgagatcggc** < 600

510 520 530 540 550 560 570 580 590

**gagcagccgtgggggcgggagttcgccctgcgcgacccggccggcaactgcgtgcacttcgtggccgaggagcaggactga**tttttttaaggcagttatt < 700

610 620 630 640 650 660 670 680 690

ggtgcccttaaacgcctggttgctacgcctgaataagtgataataagcggatgaatggcagaaattcgaaagcaaattcgacccggtcgtcggttcaggg < 800

710 720 730 740 750 760 770 780 790

cagggtcgttaaatagccgcttatgtctattgctggtttaccggtttattgactaccggaagcagtgtgaccgtgtgcttctcaaatgcctgaggccagt < 900

810 820 830 840 850 860 870 880 890

**<UR1**

|

ttgctcaggctctcccgt**gtggtgtggtgtggttgtgtg** < 939

910 920 930

Features :

UL1 : [1 : 21]

promoter : [214 : 240]

Zeocin resistance CDS : [307 : 681]

UR1 : [939 : 919]

# Gentamycin Cassette

**>UL1**

|

**caccaaacaccccccaaaacc**aagccactggagcacctcaaaaacaccatcatacactaaatcagtaagttggcagcaccacccgacgcactttgcgccg < 100

10 20 30 40 50 60 70 80 90

aataaatacctgtgacggaagatcacttcgcagaataaataaatcctggtgtccctgttgataccgggaagccctgggccaacttttggcgaaaatgaga < 200

110 120 130 140 150 160 170 180 190

**>promoter**

|

cgttgatcggcac**gtaagaggttccaactttcaccataat**gaaataagatcactaccgggcgtattttttgagttatcgagattttcaggagctaaggaa < 300

210 220 230 240 250 260 270 280 290

gctaaa**atgttacgcagcagcaacgatgttacgcagcagggcagtcgccctaaaacaaagttaggtggctcaagtatgggcatcattcgcacatgtaggc** < 400

310 320 330 340 350 360 370 380 390

**tcggccctgaccaagtcaaatccatgcgggctgctcttgatcttttcggtcgtgagttcggagacgtagccacctactcccaacatcagccggactccga** < 500

410 420 430 440 450 460 470 480 490

**>Gentamicin 3'-acetyltransferase CDS**

|

**ttacctcgggaacttgctccgtagtaagacattcatcgcgcttgctgccttcgaccaagaagcggttgttggcgctctcgcggcttacgttctgcccaag** < 600

510 520 530 540 550 560 570 580 590

**tttgagcagccgcgtagtgagatctatatctatgatctcgcagtctccggcgagcaccggaggcagggcattgccaccgcgctcatcaatctcctcaagc** < 700

610 620 630 640 650 660 670 680 690

**atgaggccaacgcgcttggtgcttatgtgatctacgtgcaagcagattacggtgacgatcccgcagtggctctctatacaaagttgggcatacgggaaga** < 800

710 720 730 740 750 760 770 780 790

**agtgatgcactttgatatcgacccaagtaccgccacctaa**tttttttaaggcagttattggtgcccttaaacgcctggttgctacgcctgaataagtgat < 900

810 820 830 840 850 860 870 880 890

aataagcggatgaatggcagaaattcgaaagcaaattcgacccggtcgtcggttcagggcagggtcgttaaatagccgcttatgtctattgctggtttac < 1000

910 920 930 940 950 960 970 980 990

**<UR1**

|

cggtttattgactaccggaagcagtgtgaccgtgtgcttctcaaatgcctgaggccagtttgctcaggctctcccgt**gtggtgtggtgtggttgtgtg** < 1098

1010 1020 1030 1040 1050 1060 1070 1080 1090

Features :

UL1 : [1 : 21]

promoter : [214 : 240]

Gentamicin 3'-acetyltransferase CDS : [307 : 840]

UR1 : [1098 : 1078]

# Rifampicin Cassette

**>UL1**

|

**caccaaacaccccccaaaacc**aagccactggagcacctcaaaaacaccatcatacactaaatcagtaagttggcagcatcacccgacgcactttgcgccg < 100

10 20 30 40 50 60 70 80 90

aataaatacctgtgacggaagatcacttcgcagaataaataaatcctggtgtccctgttgataccgggaagccctgggccaacttttggcgaaaatgaga < 200

110 120 130 140 150 160 170 180 190

**>promoter**

|

cgttgatcggcac**gtaagaggttccaactttcaccataat**gaaataagatcactaccgggcgtattttttgagttatcgagttcaggagctaaggagcta < 300

210 220 230 240 250 260 270 280 290

aa**atggtaaaagattggattcccatctctcatgataattacaagcaggtgcaaggaccgttctatcatggaaccaaagccaatttggcgattggtgactt** < 400

310 320 330 340 350 360 370 380 390

**gctaaccacagggttcatctctcatttcgaggacggtcgtattcttaagcacatctacttttcagccttgatggagccagcagtttggggagctgaactt** < 500

410 420 430 440 450 460 470 480 490

**>Rifampicin ADP-ribosylatin transferase CDS**

|

**gctatgtcactgtctggcctcgagggtcgcggctacatatacatagttgagccaacaggaccgttcgaagacgatccgaatcttacgaacaaaaaatttc** < 600

510 520 530 540 550 560 570 580 590

**ccggtaatccaacacagtcctatagaacctgcgaacccttgagaattgttggcgttgttgaagactgggaggggcatcctgttgaattaataaggggaat** < 700

610 620 630 640 650 660 670 680 690

**gttggattcgttagaggacttaaagcgccgtggtttacacgtcattgaagactag**tttttttaaggcagttattggtgcccttaaacgcctggttgctac < 800

710 720 730 740 750 760 770 780 790

gcctgaataagtgataataagcggatgaatggcagaaattcgaaagcaaattcgacccggtcgtcggttcagggcagggtcgttaaatagccgcttatgt < 900

810 820 830 840 850 860 870 880 890

ctattgctggtttaccggtttattgactaccggaagcagtgtgaccgtgtgcttctcaaatgcctgaggccagtttgctcaggctctccccgt**gtggtgt** < 1000

910 920 930 940 950 960 970 980 990

**<UR1**

|

**ggtgtggttgtgtg** < 1014

1010

Features :

UL1 : [1 : 21]

promoter : [214 : 240]

Rifampicin ADP-ribosylatin transferase CDS : [303 : 755]

UR1 : [1014 : 994]

# pDEL vector

cgtttaagggcaccaataactgcagcgctagtacggttatccacagaatcaggggataacgcaggaaagaacatgtgagat**cagcagttcaacctgttga** < 100

10 20 30 40 50 60 70 80 90

**tagtacgtactaagctctcatgtttcacgtactaagctctcatgtttaacgtactaagctctcatgtttaacgaactaaaccctcatggctaacgtacta** < 200

110 120 130 140 150 160 170 180 190

**>R6K ori rep origin**

|

**agctctcatggctaacgtactaagctctcatgtttcacgtactaagctctcatgtttgaacaataaaattaatataaatcagcaacttaaatagcctcta** < 300

210 220 230 240 250 260 270 280 290

**aggttttaagttttataagaaaaaaaagaatatataaggcttttaaagctagcttttaaggtttaacggttgtggacaacaagccagggatgtaacgcac** < 400

310 320 330 340 350 360 370 380 390

**tgagaagcccttagagcctctcaaagcaattttgagtgacacaggaacacttaacggctgaca**tggccttagaaggcgttttagaagccctaatggatgg < 500

410 420 430 440 450 460 470 480 490

**<oDO_077**  **>oDO_076**

| |

agtcaaaacaaactagcgatctgacgct**cagtggaacgaaaactcac**gtt**aagggattttggtcattg**taattctccatggtaattcacgacggaggcca < 600

510 520 530 540 550 560 570 580 590

**<Terminator BBa-1002**

**>Terminator BBa-1002**  **>Terminator BBa-1004**

| |

c**ctcatgttagtcatgcgcaaaaaaccccgcttcggcggggttttttcgcgcccgccgaaaaccccgcttcggcggggttttgccgctcagctactaggg** < 700

610 620 630 640 650 660 670 680 690

**>I-Scei binding site**  **>pRHA promoter**

| |

**ataacagggtaatgaccaggtccttcagaacgttcagcaccacaattcagcaaattgtgaacatcatcacgttcatctttccctggttgccaatggccca** < 800

710 720 730 740 750 760 770 780 790

**ttttcctgtcagtaacgagaaggtcgcgaattcaggcgctttttagactggtcgtagtttaactttaaggaggggatctatgaagaacatcaaaaagaat** < 900

810 820 830 840 850 860 870 880 890

**caagtaatcaatttcggacctaattctaaattattaaaagaatataaatcacaattaattgaattaaatattgaacaatttgaagcaggtattggtttaa** < 1000

910 920 930 940 950 960 970 980 990

**>I-SceI gBlock**

|

**ttttaggagatgcttatattcgtagtcgtgatgaaggtaaactatattgtatgcaatttgagtggaaaaataaggcatacatggatcatgtatgtttatt** < 1100

1010 1020 1030 1040 1050 1060 1070 1080 1090

**atatgatcaatgggtattatcacctcctcataaaaaagaaagagttaatcatttaggtaatttagtaattacctggggagctcaaactttcaaacatcaa** < 1200

1110 1120 1130 1140 1150 1160 1170 1180 1190

**>I-SceI gene**

|

**gcttttaataaattggctaacttattcattgtaaataacaaaaagcttattcctaacaatttagttgaaaattacttgacacctataagtttggcatact** < 1300

1210 1220 1230 1240 1250 1260 1270 1280 1290

**ggtttatagatgatggaggtaaatgggattacaataaaaactctcttaataaaagtatcgtattaaatacacaaagttttactttcgaagaagtagaata** < 1400

1310 1320 1330 1340 1350 1360 1370 1380 1390

**tttggttaaaggtttaagaaataaattccaattgaattgttatgttaaaatcaataaaaacaaaccaatcatctacattgattctataagttatttaatc** < 1500

1410 1420 1430 1440 1450 1460 1470 1480 1490

**ttttataatttgatcaaaccttacttaatccctcaaatgatatacaaattacctaatactatctcatccgaaactttcttgaaataa**catggcgatagct < 1600

1510 1520 1530 1540 1550 1560 1570 1580 1590

**<oDO_069**

**>oDO_072**  **>NeoKan Promoter**

| | |

agac**tgggcggttttatggacagcaagc**gaa**ccggaattgccagctggggcgccctctggtaaggttgggaagccctgcaa**agtaaactggatggctttc < 1700

1610 1620 1630 1640 1650 1660 1670 1680 1690

ttgccgccaaggatctgatggcgcaggggatcaagatctgatcaagagacaggatgaggatcgtttcgc**atgattgaacaagatggattgcacgcaggtt** < 1800

1710 1720 1730 1740 1750 1760 1770 1780 1790

**ctccggccgcttgggtggagaggctattcggctatgactgggcacaacagacaatcggctgctctgatgccgccgtgttccggctgtcagcgcaggggcg** < 1900

1810 1820 1830 1840 1850 1860 1870 1880 1890

**cccggttctttttgtcaagaccgacctgtccggtgccctgaatgaactgcaggacgaggcagcgcggctatcgtggctggccacgacgggcgttccttgc** < 2000

1910 1920 1930 1940 1950 1960 1970 1980 1990

**gcagctgtgctcgacgttgtcactgaagcgggaagggactggctgctattgggcgaagtgccggggcaggatctcctgtcatctcaccttgctcctgccg** < 2100

2010 2020 2030 2040 2050 2060 2070 2080 2090

**>Kan CDS**

|

**agaaagtatccatcatggctgatgcaatgcggcggctgcatacgcttgatccggctacctgcccattcgaccaccaagcgaaacatcgcatcgagcgagc** < 2200

2110 2120 2130 2140 2150 2160 2170 2180 2190

**acgtactcggatggaagccggtcttgtcgatcaggatgatctggacgaagagcatcaggggctcgcgccagccgaactgttcgccaggctcaaggcgcgc** < 2300

2210 2220 2230 2240 2250 2260 2270 2280 2290

**atgcccgacggcgaggatctcgtcgtgacccatggcgatgcctgcttgccgaatatcatggtggaaaatggccgcttttctggattcatcgactgtggcc** < 2400

2310 2320 2330 2340 2350 2360 2370 2380 2390

**ggctgggtgtggcggaccgctatcaggacatagcgttggctacccgtgatattgctgaagagcttggcggcgaatgggctgaccgcttcctcgtgcttta** < 2500

2410 2420 2430 2440 2450 2460 2470 2480 2490

**<oDO_073**

**>oDO_068**

| |

**cggtatcgccgctcccgattcgcagcgcatcgccttctatcgccttcttgacgagttcttctga**gcgggactctg**gggttcgaaatgaccgaccaagcga** < 2600

2510 2520 2530 2540 2550 2560 2570 2580 2590

**>CAP binding site**  **>pLac promoter - that's what's new. The promoter**

| |

catcataacggttctggcaaatattctggcaattaat**gtgagttagctca**ctcattaggcaccccaggc**tttacactttatgcttccggctcgtatgtt**g < 2700

2610 2620 2630 2640 2650 2660 2670 2680 2690

tgtggaattgtgagcggataacaatttcacacaggaaacagccagtccgtttaggtgttttcacgagcaattgaccaacaggaggatctggaa**atgaaca** < 2800

2710 2720 2730 2740 2750 2760 2770 2780 2790

**tcaaaaagtttgcaaaacaagcaacagtattaacctttactaccgcactgctggcaggaggcgcaactcaagcgtttgcgaaagaaacgaaccaaaagcc** < 2900

2810 2820 2830 2840 2850 2860 2870 2880 2890

**atataaggaaacatacggcatttcccatattacacgccatgatatgctgcaaatccctgaacagcaaaaaaatgaaaaatatcaagttcctgagttcgat** < 3000

2910 2920 2930 2940 2950 2960 2970 2980 2990

**tcgtccacaattaaaaatatctcttctgcaaaaggcctggacgtttgggacagctggccattacaaaacgctgacggcactgtcgcaaactatcacggct** < 3100

3010 3020 3030 3040 3050 3060 3070 3080 3090

**accacatcgtctttgcattagccggagatcctaaaaatgcggatgacacatcgatttacatgttctatcaaaaagtcggcgaaacttctattgacagctg** < 3200

3110 3120 3130 3140 3150 3160 3170 3180 3190

**gaaaaacgctggccgcgtctttaaagacagcgacaaattcgatgcaaatgattctatcctaaaagaccaaacacaagaatggtcaggttcagccacattt** < 3300

3210 3220 3230 3240 3250 3260 3270 3280 3290

**acatctgacggaaaaatccgtttattctacactgatttctccggtaaacattacggcaaacaaacactgacaactgcacaagttaacgtatcagcatcag** < 3400

3310 3320 3330 3340 3350 3360 3370 3380 3390

**acagctctttgaacatcaacggtgtagaggattataaatcaatctttgacggtgacggaaaaacgtatcaaaatgtacagcagttcatcgatgaaggcaa** < 3500

3410 3420 3430 3440 3450 3460 3470 3480 3490

**>SacB CDS**

|

**ctacagctcaggcgacaaccatacgctgagagatcctcactacgtagaagataaaggccacaaatacttagtatttgaagcaaacactggaactgaagat** < 3600

3510 3520 3530 3540 3550 3560 3570 3580 3590

**ggctaccaaggcgaagaatctttatttaacaaagcatactatggcaaaagcacatcattcttccgtcaagaaagtcaaaaacttctgcaaagcgataaaa** < 3700

3610 3620 3630 3640 3650 3660 3670 3680 3690

**aacgcacggctgagttagcaaacggcgctctcggtatgattgagctaaacgatgattacacactgaaaaaagtgatgaaaccgctgattgcatctaacac** < 3800

3710 3720 3730 3740 3750 3760 3770 3780 3790

**agtaacagatgaaattgaacgcgcgaacgtctttaaaatgaacggcaaatggtatctgttcactgactcccgcggatcaaaaatgacgattgacggcatt** < 3900

3810 3820 3830 3840 3850 3860 3870 3880 3890

**acgtctaacgatatttacatgcttggttatgtttctaattctttaactggcccatacaagccgctgaacaaaactggccttgtgttaaaaatggatcttg** < 4000

3910 3920 3930 3940 3950 3960 3970 3980 3990

**atcctaacgatgtaacctttacttactcacacttcgctgtacctcaagcgaaaggaaacaatgtcgtgattacaagctatatgacaaacagaggattcta** < 4100

4010 4020 4030 4040 4050 4060 4070 4080 4090

**cgcagacaaacaatcaacgtttgcgcccagcttcctgctgaacatcaaaggcaagaaaacatctgttgtcaaagacagcatccttgaacaaggacaatta** < 4200

4110 4120 4130 4140 4150 4160 4170 4180 4190

**acagttaacaaataa** < 4215

4210

Features :

R6K ori rep origin : [82 : 463 - CW]

oDO_077 : [547 : 529 - CCW]

oDO_076 : [551 : 568 - CW]

I-SceI gBlock : [602 : 1587 - CW]

Terminator BBa-1002 : [651 : 616 - CCW]

Terminator BBa-1002 : [617 : 650 - CW]

Terminator BBa-1004 : [654 : 687 - CW]

I-Scei binding site : [696 : 713 - CW]

pRHA promoter : [738 : 856 - CW]

I-SceI gene : [880 : 1587 - CW]

oDO_072 : [1605 : 1624 - CW]

oDO_069 : [1628 : 1610 - CCW]

NeoKan Promoter : [1632 : 1681 - CW]

Kan CDS : [1770 : 2564 - CW]

oDO_068 : [2576 : 2591 - CW]

oDO_073 : [2600 : 2581 - CCW]

CAP binding site : [2638 : 2650 - CW]

pLac promoter - that's what's new. The promoter : [2670 : 2699 - CW]

SacB CDS : [2794 : 4215 - CW]

# pOD_003 – Addgene ID155362

cgtttaagggcaccaataactgcagcgctagtacggttatccacagaatcaggggataacgcaggaaagaacatgtgagat**cagcagttcaacctgttga** < 100

10 20 30 40 50 60 70 80 90

**tagtacgtactaagctctcatgtttcacgtactaagctctcatgtttaacgtactaagctctcatgtttaacgaactaaaccctcatggctaacgtacta** < 200

110 120 130 140 150 160 170 180 190

**>R6K origin of replication**

|

**agctctcatggctaacgtactaagctctcatgtttcacgtactaagctctcatgtttgaacaataaaattaatataaatcagcaacttaaatagcctcta** < 300

210 220 230 240 250 260 270 280 290

**aggttttaagttttataagaaaaaaaagaatatataaggcttttaaagctagcttttaaggtttaacggttgtggacaacaagccagggatgtaacgcac** < 400

310 320 330 340 350 360 370 380 390

**tgagaagcccttagagcctctcaaagcaattttgagtgacacaggaacacttaacggctgaca**tggccttagaaggcgttttagaagccctaatggatgg < 500

410 420 430 440 450 460 470 480 490

agtcaaaacaaactagcgatctgacgctcagtggaacgaaaactcacgttaagggattttggtcattgtaattctccatggtaattcacgacggaggcca < 600

510 520 530 540 550 560 570 580 590

**>terminator**  **>terminator**

| |

cctcatgttagtcatg**cgcaaaaaaccccgcttcggcggggttttttcgc**gcc**cgccgaaaaccccgcttcggcggggttttgccgc**tcagctac**taggg** < 700

610 620 630 640 650 660 670 680 690

**>I-SceI binding site**  **>pRHA promoter**

| |

**ataacagggtaat**gaccaggtccttcagaacgttcag**caccacaattcagcaaattgtgaacatcatcacgttcatctttccctggttgccaatggccca** < 800

710 720 730 740 750 760 770 780 790

**ttttcctgtcagtaacgagaaggtcgcgaattcaggcgctttttagactggtcgta**gtttaactttaaggaggggatct**atgaagaacatcaaaaagaat** < 900

810 820 830 840 850 860 870 880 890

**caagtaatcaatttcggacctaattctaaattattaaaagaatataaatcacaattaattgaattaaatattgaacaatttgaagcaggtattggtttaa** < 1000

910 920 930 940 950 960 970 980 990

**ttttaggagatgcttatattcgtagtcgtgatgaaggtaaactatattgtatgcaatttgagtggaaaaataaggcatacatggatcatgtatgtttatt** < 1100

1010 1020 1030 1040 1050 1060 1070 1080 1090

**atatgatcaatgggtattatcacctcctcataaaaaagaaagagttaatcatttaggtaatttagtaattacctggggagctcaaactttcaaacatcaa** < 1200

1110 1120 1130 1140 1150 1160 1170 1180 1190

**>I-SceI CDS**

|

**gcttttaataaattggctaacttattcattgtaaataacaaaaagcttattcctaacaatttagttgaaaattacttgacacctataagtttggcatact** < 1300

1210 1220 1230 1240 1250 1260 1270 1280 1290

**ggtttatagatgatggaggtaaatgggattacaataaaaactctcttaataaaagtatcgtattaaatacacaaagttttactttcgaagaagtagaata** < 1400

1310 1320 1330 1340 1350 1360 1370 1380 1390

**tttggttaaaggtttaagaaataaattccaattgaattgttatgttaaaatcaataaaaacaaaccaatcatctacattgattctataagttatttaatc** < 1500

1410 1420 1430 1440 1450 1460 1470 1480 1490

**ttttataatttgatcaaaccttacttaatccctcaaatgatatacaaattacctaatactatctcatccgaaactttcttgaaataa**catggcgatagct < 1600

1510 1520 1530 1540 1550 1560 1570 1580 1590

**>UL1**

|

agactgggcggttttatggacagcaagc**caccaaacaccccccaaaacc**aagccactggagcacctcaaaaacaccatcatacactaaatcagtaagttg < 1700

1610 1620 1630 1640 1650 1660 1670 1680 1690

gcagcaccacccgacgcactttgcgccgaataaatacctgtgacggaagatcacttcgcagaataaataaatcctggtgtccctgttgataccgggaagc < 1800

1710 1720 1730 1740 1750 1760 1770 1780 1790

**>promoter**

|

cctgggccaacttttggcgaaaatgagacgttgatcggcac**gtaagaggttccaactttcaccataat**gaaataagatcactaccgggcgtattttttga < 1900

1810 1820 1830 1840 1850 1860 1870 1880 1890

gttatcgagattttcaggagctaaggaagctaaa**atggccaagttgaccagtgccgttccggtgctcaccgcgcgcgacgtcgtcggagcggtcgagttc** < 2000

1910 1920 1930 1940 1950 1960 1970 1980 1990

**tggaccgaccggctcgggttctcccgggacttcgtggaggacgacttcgccggtgtggtccgggacgacgtgaccctgttcatcagcgcggtccaggacc** < 2100

2010 2020 2030 2040 2050 2060 2070 2080 2090

**>Zeocin resistance CDS**

|

**aggcggtgccggacaacaccctggcctgggtgtgggtgcgcggcctggacgagctgtacgccgagtggtcggaggtcgtgtccacggacttccgggacgc** < 2200

2110 2120 2130 2140 2150 2160 2170 2180 2190

**ctccgggccggccatgaccgagatcggcgagcagccgtgggggcgggagttcgccctgcgcgacccggccggcaactgcgtgcacttcgtggccgaggag** < 2300

2210 2220 2230 2240 2250 2260 2270 2280 2290

**caggactga**tttttttaaggcagttattggtgcccttaaacgcctggttgctacgcctgaataagtgataataagcggatgaatggcagaaattcgaaag < 2400

2310 2320 2330 2340 2350 2360 2370 2380 2390

caaattcgacccggtcgtcggttcagggcagggtcgttaaatagccgcttatgtctattgctggtttaccggtttattgactaccggaagcagtgtgacc < 2500

2410 2420 2430 2440 2450 2460 2470 2480 2490

**<UR1**

|

gtgtgcttctcaaatgcctgaggccagtttgctcaggctctcccgt**gtggtgtggtgtggttgtgtg**gggttcgaaatgaccgaccaagcgacatcataa < 2600

2510 2520 2530 2540 2550 2560 2570 2580 2590

**>CAP binding site_misc_feature**  **>pLac promoter**

| |

cggttctggcaaatattctggcaattaat**gtgagttagctca**ctcattaggcaccccaggc**tttacactttatgcttccggctcgtatgtt**gtgtggaat < 2700

2610 2620 2630 2640 2650 2660 2670 2680 2690

tgtgagcggataacaatttcacacaggaaacagccagtccgtttaggtgttttcacgagcaattgaccaacaggaggatctggaa**atgaacatcaaaaag** < 2800

2710 2720 2730 2740 2750 2760 2770 2780 2790

**tttgcaaaacaagcaacagtattaacctttactaccgcactgctggcaggaggcgcaactcaagcgtttgcgaaagaaacgaaccaaaagccatataagg** < 2900

2810 2820 2830 2840 2850 2860 2870 2880 2890

**aaacatacggcatttcccatattacacgccatgatatgctgcaaatccctgaacagcaaaaaaatgaaaaatatcaagttcctgagttcgattcgtccac** < 3000

2910 2920 2930 2940 2950 2960 2970 2980 2990

**aattaaaaatatctcttctgcaaaaggcctggacgtttgggacagctggccattacaaaacgctgacggcactgtcgcaaactatcacggctaccacatc** < 3100

3010 3020 3030 3040 3050 3060 3070 3080 3090

**gtctttgcattagccggagatcctaaaaatgcggatgacacatcgatttacatgttctatcaaaaagtcggcgaaacttctattgacagctggaaaaacg** < 3200

3110 3120 3130 3140 3150 3160 3170 3180 3190

**ctggccgcgtctttaaagacagcgacaaattcgatgcaaatgattctatcctaaaagaccaaacacaagaatggtcaggttcagccacatttacatctga** < 3300

3210 3220 3230 3240 3250 3260 3270 3280 3290

**cggaaaaatccgtttattctacactgatttctccggtaaacattacggcaaacaaacactgacaactgcacaagttaacgtatcagcatcagacagctct** < 3400

3310 3320 3330 3340 3350 3360 3370 3380 3390

**>SacB_CDS**

|

**ttgaacatcaacggtgtagaggattataaatcaatctttgacggtgacggaaaaacgtatcaaaatgtacagcagttcatcgatgaaggcaactacagct** < 3500

3410 3420 3430 3440 3450 3460 3470 3480 3490

**caggcgacaaccatacgctgagagatcctcactacgtagaagataaaggccacaaatacttagtatttgaagcaaacactggaactgaagatggctacca** < 3600

3510 3520 3530 3540 3550 3560 3570 3580 3590

**aggcgaagaatctttatttaacaaagcatactatggcaaaagcacatcattcttccgtcaagaaagtcaaaaacttctgcaaagcgataaaaaacgcacg** < 3700

3610 3620 3630 3640 3650 3660 3670 3680 3690

**gctgagttagcaaacggcgctctcggtatgattgagctaaacgatgattacacactgaaaaaagtgatgaaaccgctgattgcatctaacacagtaacag** < 3800

3710 3720 3730 3740 3750 3760 3770 3780 3790

**atgaaattgaacgcgcgaacgtctttaaaatgaacggcaaatggtatctgttcactgactcccgcggatcaaaaatgacgattgacggcattacgtctaa** < 3900

3810 3820 3830 3840 3850 3860 3870 3880 3890

**cgatatttacatgcttggttatgtttctaattctttaactggcccatacaagccgctgaacaaaactggccttgtgttaaaaatggatcttgatcctaac** < 4000

3910 3920 3930 3940 3950 3960 3970 3980 3990

**gatgtaacctttacttactcacacttcgctgtacctcaagcgaaaggaaacaatgtcgtgattacaagctatatgacaaacagaggattctacgcagaca** < 4100

4010 4020 4030 4040 4050 4060 4070 4080 4090

**aacaatcaacgtttgcgcccagcttcctgctgaacatcaaaggcaagaaaacatctgttgtcaaagacagcatccttgaacaaggacaattaacagttaa** < 4200

4110 4120 4130 4140 4150 4160 4170 4180 4190

**caaataa** < 4207

Features :

R6K origin of replication : [82 : 463 - CW]

terminator : [617 : 650 - CW]

terminator : [654 : 687 - CW]

I-SceI binding site : [696 : 713 - CW]

pRHA promoter : [738 : 856 - CW]

I-SceI CDS : [880 : 1587 - CW]

UL1 : [1629 : 1649 - CW]

promoter : [1842 : 1868 - CW]

Zeocin resistance CDS : [1935 : 2309 - CW]

UR1 : [2567 : 2547 - CCW]

CAP binding site_misc_feature : [2630 : 2642 - CW]

pLac promoter : [2662 : 2691 - CW]

SacB_CDS : [2786 : 4207 - CW]

# pOD_004 – Addgene ID155361

cgtttaagggcaccaataactgcagcgctagtacggttatccacagaatcaggggataacgcaggaaagaacatgtgagat**cagcagttcaacctgttga** < 100

10 20 30 40 50 60 70 80 90

**tagtacgtactaagctctcatgtttcacgtactaagctctcatgtttaacgtactaagctctcatgtttaacgaactaaaccctcatggctaacgtacta** < 200

110 120 130 140 150 160 170 180 190

**>R6K origin of replication**

|

**agctctcatggctaacgtactaagctctcatgtttcacgtactaagctctcatgtttgaacaataaaattaatataaatcagcaacttaaatagcctcta** < 300

210 220 230 240 250 260 270 280 290

**aggttttaagttttataagaaaaaaaagaatatataaggcttttaaagctagcttttaaggtttaacggttgtggacaacaagccagggatgtaacgcac** < 400

310 320 330 340 350 360 370 380 390

**tgagaagcccttagagcctctcaaagcaattttgagtgacacaggaacacttaacggctgaca**tggccttagaaggcgttttagaagccctaatggatgg < 500

410 420 430 440 450 460 470 480 490

agtcaaaacaaactagcgatctgacgctcagtggaacgaaaactcacgttaagggattttggtcattgtaattctccatggtaattcacgacggaggcca < 600

510 520 530 540 550 560 570 580 590

**>terminator**  **>terminator**

| |

cctcatgttagtcatg**cgcaaaaaaccccgcttcggcggggttttttcgc**gcc**cgccgaaaaccccgcttcggcggggttttgccgc**tcagctac**taggg** < 700

610 620 630 640 650 660 670 680 690

**>I-SceI binding site**  **>pRHA promoter**

| |

**ataacagggtaat**gaccaggtccttcagaacgttcag**caccacaattcagcaaattgtgaacatcatcacgttcatctttccctggttgccaatggccca** < 800

710 720 730 740 750 760 770 780 790

**ttttcctgtcagtaacgagaaggtcgcgaattcaggcgctttttagactggtcgta**gtttaactttaaggaggggatct**atgaagaacatcaaaaagaat** < 900

810 820 830 840 850 860 870 880 890

**caagtaatcaatttcggacctaattctaaattattaaaagaatataaatcacaattaattgaattaaatattgaacaatttgaagcaggtattggtttaa** < 1000

910 920 930 940 950 960 970 980 990

**ttttaggagatgcttatattcgtagtcgtgatgaaggtaaactatattgtatgcaatttgagtggaaaaataaggcatacatggatcatgtatgtttatt** < 1100

1010 1020 1030 1040 1050 1060 1070 1080 1090

**atatgatcaatgggtattatcacctcctcataaaaaagaaagagttaatcatttaggtaatttagtaattacctggggagctcaaactttcaaacatcaa** < 1200

1110 1120 1130 1140 1150 1160 1170 1180 1190

**>I-SceI CDS**

|

**gcttttaataaattggctaacttattcattgtaaataacaaaaagcttattcctaacaatttagttgaaaattacttgacacctataagtttggcatact** < 1300

1210 1220 1230 1240 1250 1260 1270 1280 1290

**ggtttatagatgatggaggtaaatgggattacaataaaaactctcttaataaaagtatcgtattaaatacacaaagttttactttcgaagaagtagaata** < 1400

1310 1320 1330 1340 1350 1360 1370 1380 1390

**tttggttaaaggtttaagaaataaattccaattgaattgttatgttaaaatcaataaaaacaaaccaatcatctacattgattctataagttatttaatc** < 1500

1410 1420 1430 1440 1450 1460 1470 1480 1490

**ttttataatttgatcaaaccttacttaatccctcaaatgatatacaaattacctaatactatctcatccgaaactttcttgaaataa**catggcgatagct < 1600

1510 1520 1530 1540 1550 1560 1570 1580 1590

**>UL1**

|

agactgggcggttttatggacagcaagc**caccaaacaccccccaaaacc**aagccactggagcacctcaaaaacaccatcatacactaaatcagtaagttg < 1700

1610 1620 1630 1640 1650 1660 1670 1680 1690

gcagcaccacccgacgcactttgcgccgaataaatacctgtgacggaagatcacttcgcagaataaataaatcctggtgtccctgttgataccgggaagc < 1800

1710 1720 1730 1740 1750 1760 1770 1780 1790

**>promoter**

|

cctgggccaacttttggcgaaaatgagacgttgatcggcac**gtaagaggttccaactttcaccataat**gaaataagatcactaccgggcgtattttttga < 1900

1810 1820 1830 1840 1850 1860 1870 1880 1890

gttatcgagattttcaggagctaaggaagctaaa**atgttacgcagcagcaacgatgttacgcagcagggcagtcgccctaaaacaaagttaggtggctca** < 2000

1910 1920 1930 1940 1950 1960 1970 1980 1990

**agtatgggcatcattcgcacatgtaggctcggccctgaccaagtcaaatccatgcgggctgctcttgatcttttcggtcgtgagttcggagacgtagcca** < 2100

2010 2020 2030 2040 2050 2060 2070 2080 2090

**cctactcccaacatcagccggactccgattacctcgggaacttgctccgtagtaagacattcatcgcgcttgctgccttcgaccaagaagcggttgttgg** < 2200

2110 2120 2130 2140 2150 2160 2170 2180 2190

**>Gentamicin 3'-acetyltransferase CDS**

|

**cgctctcgcggcttacgttctgcccaagtttgagcagccgcgtagtgagatctatatctatgatctcgcagtctccggcgagcaccggaggcagggcatt** < 2300

2210 2220 2230 2240 2250 2260 2270 2280 2290

**gccaccgcgctcatcaatctcctcaagcatgaggccaacgcgcttggtgcttatgtgatctacgtgcaagcagattacggtgacgatcccgcagtggctc** < 2400

2310 2320 2330 2340 2350 2360 2370 2380 2390

**tctatacaaagttgggcatacgggaagaagtgatgcactttgatatcgacccaagtaccgccacctaa**tttttttaaggcagttattggtgcccttaaac < 2500

2410 2420 2430 2440 2450 2460 2470 2480 2490

gcctggttgctacgcctgaataagtgataataagcggatgaatggcagaaattcgaaagcaaattcgacccggtcgtcggttcagggcagggtcgttaaa < 2600

2510 2520 2530 2540 2550 2560 2570 2580 2590

tagccgcttatgtctattgctggtttaccggtttattgactaccggaagcagtgtgaccgtgtgcttctcaaatgcctgaggccagtttgctcaggctct < 2700

2610 2620 2630 2640 2650 2660 2670 2680 2690

**<UR1**  **>CAP binding site_misc_feature**

| |

cccgt**gtggtgtggtgtggttgtgtg**gggttcgaaatgaccgaccaagcgacatcataacggttctggcaaatattctggcaattaat**gtgagttagctc** < 2800

2710 2720 2730 2740 2750 2760 2770 2780 2790

**>promoter**

|

**a**ctcattaggcaccccaggc**tttacactttatgcttccggctcgtatgtt**gtgtggaattgtgagcggataacaatttcacacaggaaacagccagtccg < 2900

2810 2820 2830 2840 2850 2860 2870 2880 2890

tttaggtgttttcacgagcaattgaccaacaggaggatctggaa**atgaacatcaaaaagtttgcaaaacaagcaacagtattaacctttactaccgcact** < 3000

2910 2920 2930 2940 2950 2960 2970 2980 2990

**gctggcaggaggcgcaactcaagcgtttgcgaaagaaacgaaccaaaagccatataaggaaacatacggcatttcccatattacacgccatgatatgctg** < 3100

3010 3020 3030 3040 3050 3060 3070 3080 3090

**caaatccctgaacagcaaaaaaatgaaaaatatcaagttcctgagttcgattcgtccacaattaaaaatatctcttctgcaaaaggcctggacgtttggg** < 3200

3110 3120 3130 3140 3150 3160 3170 3180 3190

**acagctggccattacaaaacgctgacggcactgtcgcaaactatcacggctaccacatcgtctttgcattagccggagatcctaaaaatgcggatgacac** < 3300

3210 3220 3230 3240 3250 3260 3270 3280 3290

**atcgatttacatgttctatcaaaaagtcggcgaaacttctattgacagctggaaaaacgctggccgcgtctttaaagacagcgacaaattcgatgcaaat** < 3400

3310 3320 3330 3340 3350 3360 3370 3380 3390

**gattctatcctaaaagaccaaacacaagaatggtcaggttcagccacatttacatctgacggaaaaatccgtttattctacactgatttctccggtaaac** < 3500

3410 3420 3430 3440 3450 3460 3470 3480 3490

**attacggcaaacaaacactgacaactgcacaagttaacgtatcagcatcagacagctctttgaacatcaacggtgtagaggattataaatcaatctttga** < 3600

3510 3520 3530 3540 3550 3560 3570 3580 3590

**>SacB_CDS**

|

**cggtgacggaaaaacgtatcaaaatgtacagcagttcatcgatgaaggcaactacagctcaggcgacaaccatacgctgagagatcctcactacgtagaa** < 3700

3610 3620 3630 3640 3650 3660 3670 3680 3690

**gataaaggccacaaatacttagtatttgaagcaaacactggaactgaagatggctaccaaggcgaagaatctttatttaacaaagcatactatggcaaaa** < 3800

3710 3720 3730 3740 3750 3760 3770 3780 3790

**gcacatcattcttccgtcaagaaagtcaaaaacttctgcaaagcgataaaaaacgcacggctgagttagcaaacggcgctctcggtatgattgagctaaa** < 3900

3810 3820 3830 3840 3850 3860 3870 3880 3890

**cgatgattacacactgaaaaaagtgatgaaaccgctgattgcatctaacacagtaacagatgaaattgaacgcgcgaacgtctttaaaatgaacggcaaa** < 4000

3910 3920 3930 3940 3950 3960 3970 3980 3990

**tggtatctgttcactgactcccgcggatcaaaaatgacgattgacggcattacgtctaacgatatttacatgcttggttatgtttctaattctttaactg** < 4100

4010 4020 4030 4040 4050 4060 4070 4080 4090

**gcccatacaagccgctgaacaaaactggccttgtgttaaaaatggatcttgatcctaacgatgtaacctttacttactcacacttcgctgtacctcaagc** < 4200

4110 4120 4130 4140 4150 4160 4170 4180 4190

**gaaaggaaacaatgtcgtgattacaagctatatgacaaacagaggattctacgcagacaaacaatcaacgtttgcgcccagcttcctgctgaacatcaaa** < 4300

4210 4220 4230 4240 4250 4260 4270 4280 4290

**ggcaagaaaacatctgttgtcaaagacagcatccttgaacaaggacaattaacagttaacaaataa** < 4366

4310 4320 4330 4340 4350 4360

**Features :**

**R6K origin of replication : [82 : 463 - CW]**

**terminator : [617 : 650 - CW]**

**terminator : [654 : 687 - CW]**

**I-SceI binding site : [696 : 713 - CW]**

**pRHA promoter : [738 : 856 - CW]**

**I-SceI CDS : [880 : 1587 - CW]**

**UL1 : [1629 : 1649 - CW]**

**promoter : [1842 : 1868 - CW]**

**Gentamicin 3'-acetyltransferase CDS : [1935 : 2468 - CW]**

**UR1 : [2726 : 2706 - CCW]**

**CAP binding site_misc_feature : [2789 : 2801 - CW]**

**promoter : [2821 : 2850 - CW]**

**SacB_CDS : [2945 : 4366 - CW]**
