# Supplementary material for: Reassembling a cannon in the DNA defense arsenal: Genetics of StySA, a BREX phage exclusion system in Salmonella lab strains
Source: PLoS Genet. 2022 Apr 4;18(4):e1009943. doi: 10.1371/journal.pgen.1009943 (PMC9009780; doi:10.1371/journal.pgen.1009943)
Supplement: S6 File — This includes three figures, an annotation protocol, and extended discussion of activities of other proteins with domain hits like that of the BrxL C-terminal domain. (DOCX) [file pgen.1009943.s009.docx]

**Domain search: examples BrxC, BrxL**

Implemented in:

Geneious: Geneious Prime 2021.1.1
Build 2021-03-12 13:25

**I. IPRScan use protocol**

Used the Protein IDentifiers of S1 Table to

1. download amino acid sequences from NCBI>Proteins>Protein Database to Geneious
   1. folder "Proteins", subfolder "StySA proteins domain search recapitulation"
2. NCBI file comes with annotations
   1. "CDS" with "locus_tag" identificaton
   2. NCBI Feature Key "Protein" with "product", which can be a "DUF", domain of unknown function (or more elaborate description)
   3. NCBI Feature Key "Region" with Conserved Domain Database reference, "CDD:number"
3. Annotate from InterProScan facility
   1. Choose Annotate and Predict submenu
   2. Choose "Find Protein Domains with InterPro Scan"
   3. Check add features to proteins without Interpro results or with errors
   4. Show as qualifiers
   5. Check domain annotation sources
      1. Panther
      2. TigrFam
      3. Superfamily
      4. Prosite Profiles
      5. PfamA
      6. Gene3d
      7. not picked: ProDom, PRINTS, PIRSF, SMART, HAMAP, PrositePatterns, SignalP, TMHMM, Phobius

Annotation Data reference files with complete domain annotation downloaded, example of BrxC:

1. In Sequence View, Annotations tab
   1. check "Show Annotations"
2. In Annotations Table (below the sequence view)
   1. click "Columns"
   2. If necessary click "Manage columns" to add fields to the table
   3. check fields for display in table. These columns can be rearranged by dragging. Below is the order I chose. Not all annotations have an entry in every column
      1. Name (this is the name of the annotation)
      2. Type
      3. Minimum (aa coordinate)
      4. Maximum
      5. Length
      6. # intervals
      7. Document Name
      8. Sequence name
      9. locus_tag
      10. NCBI Feature
      11. Note
      12. product
      13. Database
      14. Id (this is a domain Id number)
      15. Name (of domain)
      16. InterPro ID
      17. InterPro Name
      18. InterPro Type
      19. Created by (for variants)
   4. click "Export Table"
   5. choose .csv or.tsv; I chose .csv
   6. Rename the table as convenient. I added "IPRscan" to the default name.
3. Exported table: e.g. IPRscan BrxC ER3625-QTP99674.1.csv
4. For visualization, made alignments (BrxC, PglZ) or illustrative figures.
   1. deleted from the table or unchecked annotations that covered the entire protein (redundant information)
   2. Exported table with CDS and remaining domain annotations
   3. Alignment reference files exported with the same procedure as individual proteins

**II. BrxL Domain annotations**

STM4491 (JJB80_22575) is a BrxL homolog. Domain predictions for protein sequence AAL23309 (NP_463350.1) gave multiple hits to Lon-protease domains. This is proposed to be two-domain Lon-like protease structure with an N-terminal ATPase and a C-terminal proteolytic domain. However, it is most likely not a protease as the catalytic site is not present (see further below).

additional annotations divide the protein into at least two segments. The N-terminal region of BrxL draws annotations related to AAA+ NTPase activity, while the C-terminal domain draws separate annotations, some related to protease function, others more general. The enzymatic activity of the C-terminal domain is of particular interest.

Domains of BrxL protein QTP99671.1 predicted by online domain finders from Geneious-enabled IPRScan obtained as described in"I. IPRScan use protocol" above. Top line: amino acid coordinates; blue boxes: TIGRFAM annotations; yellow box: GENE3d:3:30 Ribosomal_S5_D2-typ_fold_subgr; black box: annotation of locus_tag JJB80_22575; purple boxes: PFAM annotations; dark green boxes: Superfamily SSF52540 (P-loop_NTPase) and SSF54211 (Ribosomal_S5_D2-typ_fold).

**III. Proteins of LT2 that draw annotations similar to those of the BrxL C-terminus**

This was carried out as described below (IV). Three of four annotations covering the C-terminal region suggest that BrxL is not necessarily a protease:

A. TIGR02653: "Lon-rel chp" [chp is "conserved hypothetical protein"] describes this region in CDD [1], specifically calling attention to the lack of catalytic residues involved in protease activity: on 19 July 21:

**conserved hypothetical protein**

This model describes a protein family of unknown function, about 690 residues in length, in which some members show C-terminal sequence similarity to pfam05362, which is the Lon protease C-terminal proteolytic domain, from MEROPS family S16. However, the annotated catalytic sites of E. coli Lon protease are not conserved in members of this family. Members have a motif GP[RK][GS]TGKS, similar to the ATP-binding P-loop motif GxxGxGK[ST]. [Hypothetical proteins, Conserved]

B. PF05362: "Lon protease (S16) C-terminal proteolytic domain"

This domain is found three times in S. enterica sv Typhimurium LT2, searching Uniprot as described below. Two are in regions extensively syntenic with *E. coli*: *lon* (STM0450, b0439) and *lonH* (STM1068; *E. coli ycbZ* b0955). The third is *brxL* (STM4491), in the *leuX* mobile island. Some *E. coli* strains carry a BREX related system at this locus, but many don't. Essentially all serovar Typhiumurium do carry StySA. Note that catalytic proteolysis residues are specifically not conserved in hits to TIGR0653 above. Not much is known about LonH.

From Pfam, Interpro annotation; description of catalytic site in bold:

Lon (also known as endopeptidase La) is a multi-domain ATP- dependent protease found throughout all kingdoms of life. It is involved in protein quality control and several regulatory processes. All Lon proteases contain an ATPase domain belonging to the AAA+ superfamily of molecular machines, and a proteolytic domain with a serine-lysine catalytic dyad **in which a lysine assists the catalytic serine in proteolytic cleavage**. Lon proteases can be divided into two subfamilies: A type (A-Lons), which have a large multi-lobed N-terminal domain together with the ATPase and protease domains, and B type (B-Lons), which lack an N domain, but have a membrane-anchoring region emerging from the ATPase domain. B-Lons are found in Archaea, in which they are the lone membrane-anchored ATP-dependent protease. The soluble A-Lons are found in all bacteria and in eukaryotic cell organelles, such as mitochondria and peroxisomes, and are needed for recovery from various stress conditions (references). The Lon proteolytic domain forms peptidase family S16 of clan SJ (ref). The structure of the Lon proteolytic domain consists of six alpha helices and ten beta strands

C. Superfamily SSF54211: "Ribosomal_S5_D2-typ_fold" draws attention to other potential functions for the C-terminal domain besides proteolysis. This domain is found in 22 LT2 proteins, acting on DNA, RNA and small molecule substrates as well as proteins. The list* was obtained as in IV.

22 proteins are identified, of which: 7 act on nucleic acids (e.g. RadA, DNA gyrase, MutL, RNaseP); 6 on small molecules (kinases especially, e.g. threonine kinase, galactokinase) and 7 proteins (Lon, LonH, EF-G, RpsI) by my classification. BrxL and uncharacterized protein YifB I did not classify. Enzymatic categorization by Uniprot summarizes the Enzyme Commission categorization as: 6 transfer phosphorus-containing groups (E.C. 2.7.-.-); 6 hydrolases (E.C. 3.-.-.-); one dehydratase (E.C. 4.2.1.19); 2 DNA topoisomerase (ATP-hydrolyzing) (E.C. 5.6.2.2). Those not addressed by the Enzyme Commission include ribosome components, a chaperone and unknown functions.

D. Gene3D category 3.30.230.10 (Ribosomal_S5_D2-typ_fold_subgr) retrieves 15 of the 22 proteins of SSF54211, omitting some from each group†. All of those listed above for SSF54211 are detected.

**IV. Identifying other LT2 proteins with related C-terminal domains using domain search**

- At Uniprot: https://www.uniprot.org/
- Salmonella typhimurium (strain LT2 / SGSC1412 / ATCC 700720 is organism 99287.
- search "SSF54211 AND organism: 99287"
- select all entries
- View by results table
- 12 Columns:
  - Entry (code)
  - Entry name (mnemonic)
  - reviewed or not
  - protein names (incl EC number)
  - gene names (gene and locus_ID)
  - organism
  - length
  - 5 columns of GO terms
  - three clickable text (biological process, cellular component, molecular function; clicking goes to GONUTS page)
  - two combining entries from the three categories, one as clickable text, the other with the corresponding GO:numbers (also clickable).
- Download: click Download tab
- Choose from menu: download all
- Choose format: Excel (8 other options exist)
- Choose Uncompressed (I couldn't use the compressed version, but this is a small list)
- hit Go

File is named: "uniprot-SSF54211+++organism_+99287.xlsx"

Repeat with

- PF05362 AND organism:99287
- 3.30.230.10 AND organism:99287

Additional characteristics of the SF54211hits, in file¶, give Enzyme Commission number, catalytic activity, DNA binding (none listed) and Biocyc cross reference (only one, threonine kinase)

Shareable link: https://www.uniprot.org/uniprot/?query=SSF54211%20%2B%20organism%3A%2099287&columns=id%2Centry%20name%2Creviewed%2Cprotein%20names%2Cgenes%2Corganism%2Clength%2Cgo(biological%20process)%2Cgo(cellular%20component)%2Cgo%2Cgo(molecular%20function)%2Cgo-id&sort=score

Also available at this page: categorization by taxonomy, keywords, gene ontology, E.c. class, pathway.

Output files referred to but not included.

*LT2 SSFlonc uniprot-organism__Salmonella+typhimurium+(strain+LT2+_+SGSC1412+_+ATCC--.xlsx

†LT2 g3d lon uniprot-gene3d+3.30.230.10+AND+organism__Salmonella+typhimurium+(strain+LT--.xlsx

§S5_File RNAseq_genome_hits.xlsx

¶ uniprot-SSF54211+++organism_+99287 ECno

**V. Potential BrxL activities**

An important aspect of BREX phage restriction (observed by others for related systems) is very early interruption of development. The infecting phage injects DNA but does not replicate and is not degraded [2]. BrxL is specifically required for restriction (reduction in plaque formation), but not modification [3]. BrxL action should thus interrupt a very early event in infection (likely targeted by other components StySA complex). Phosphorylation of the 3' end of a replication fork or replication primer (a sugar alcohol) or covalent attachment to DNA (as for topoisomerases) could account for the failure to replicate of sensitive phages. The kinase and topoisomerase activities found for some examples of this domain are of thus of particular interest. The inferred phosphatase activity of PglZ might pair with a ('BrxL) kinase activity in the BREX cluster, e.g. to reverse a toxic product.

The four candidate activities of the C-terminal domain: hydrolysis, phosphoryl transfer and folding each could explain the curious fact that all three *∆brxL* strains share a severe disruption of cellular metabolism (see S5 File). High transcription driven by p*cat* is likely to lead to translation of an in-frame solo C-terminal domain in the three *∆brxL* constructs. In these, a *cat* cassette with a highly active promoter but no transcription terminator has replaced 1278 nt (of 2085) in the 5' region of *brxL*. 707 bases of the 3' end of *brxL* plus remain. A transcript from p*cat* of ~1435 nt has frames for CAT and an in-frame fragment of BrxL ('BrxL, 669 nt) (see below). No transcription increase affects the neighboring gene (*mrr2*) due to strong terminator insulation (see Fig 5B main text)

DNA configuration in a *brxL::cat* strain. The translation of the original *brxL* CDS (STM4491) terminates within the added *cat* cassette (118 nt + 10 nt cassette; 43aa). A translation restart at CTG 20 nt into the *brxL* remnant (52 nt from the *cat* UGA), would lead to expression of a protein carrying the signature motifs below. Yellow arrows: CDS; blue arrows: remaining fragments of the original CDS annotation.

Protein domain annotations for predicted C-terminal fragment. yellow box: GENE3d:3:30 Ribosomal_S5_D2-typ_fold_subgr; purple boxes: PFAM annotations; dark green box: Superfamily SSF54211 (Ribosomal_S5_D2-typ_fold).

Substantial transcription of remaining *'brxL* transcript is indeed found by RNAseq (see Fig 5B main text).

Growth rate is impaired when *∆L* is present in a *Z^+^C^+^* context (Fig 4 main text) but not impaired when in the context of either *Zµ* or *Cµ*. In all three cases two of the three prophages in the genome express structural proteins but not replication functions (S4 file). The genome-wide effects could be due to translation of a protein domain lacking the control on its action that might be afforded by association with the other proteins in the BREX cluster.

LITERATURE CITED

1. Lu S, Wang J, Chitsaz F, Derbyshire MK, Geer RC, Gonzales NR, et al. CDD/SPARCLE: the conserved domain database in 2020. Nucleic Acids Research. 2019;48(D1):D265-D8. doi: 10.1093/nar/gkz991 PMID - 31777944.

2. Goldfarb T, Sberro H, Weinstock E, Cohen O, Doron S, Charpak-Amikam Y, et al. BREX is a novel phage resistance system widespread in microbial genomes. EMBO J. 2015;34(2):169-83. Epub 2014/12/03. doi: 10.15252/embj.201489455. PubMed PMID: 25452498; PubMed Central PMCID: PMCPMC4337064.

3. Gordeeva J, Morozova N, Sierro N, Isaev A, Sinkunas T, Tsvetkova K, et al. BREX system of Escherichia coli distinguishes self from non-self by methylation of a specific DNA site. Nucleic Acids Res. 2019;47(1):253-65. Epub 2018/11/13. doi: 10.1093/nar/gky1125. PubMed PMID: 30418590; PubMed Central PMCID: PMCPMC6326788.
