# Supplementary material for: Reassembling a cannon in the DNA defense arsenal: Genetics of StySA, a BREX phage exclusion system in Salmonella lab strains
Source: PLoS Genet. 2022 Apr 4;18(4):e1009943. doi: 10.1371/journal.pgen.1009943 (PMC9009780; doi:10.1371/journal.pgen.1009943)

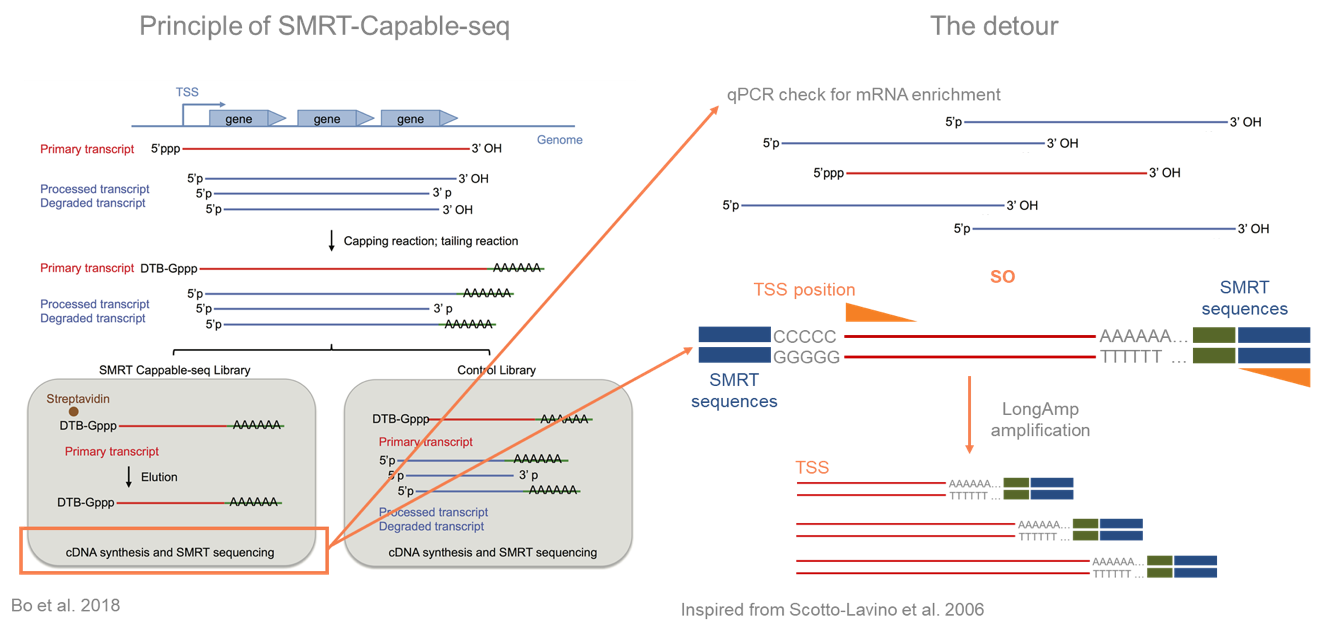
Panel A: The method
SMRT-recovered transcripts (5' end enrichment) tailed with polyA-polymerase (3' end enrichment) were then used for qPCR

Using the method to characterize transcription at the StySA-BREX locus of LT2 (STK013)

Panel B: SMRT-recovered transcripts initiating at brxA

The blue rectangles correspond to CDS and the orange triangles to the Cappable-seq primers.


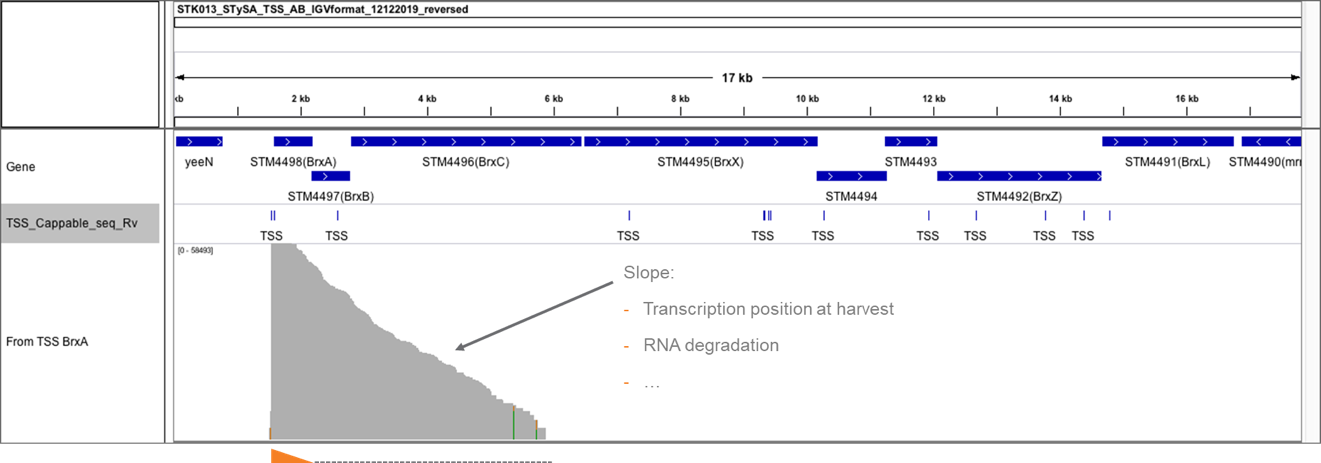


Panel C. Closeup of reads of panel B (brxA TSS) near the candidate *brxC* terminator


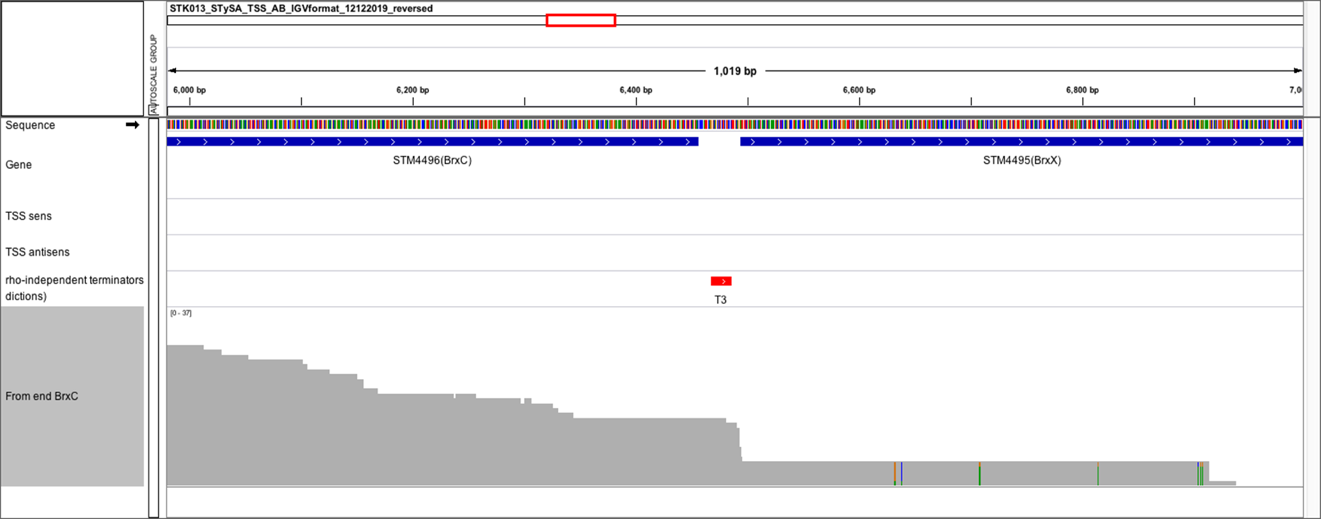


Panel D. SMRT-recovered transcripts initiating inside of STM4494 and STM4493


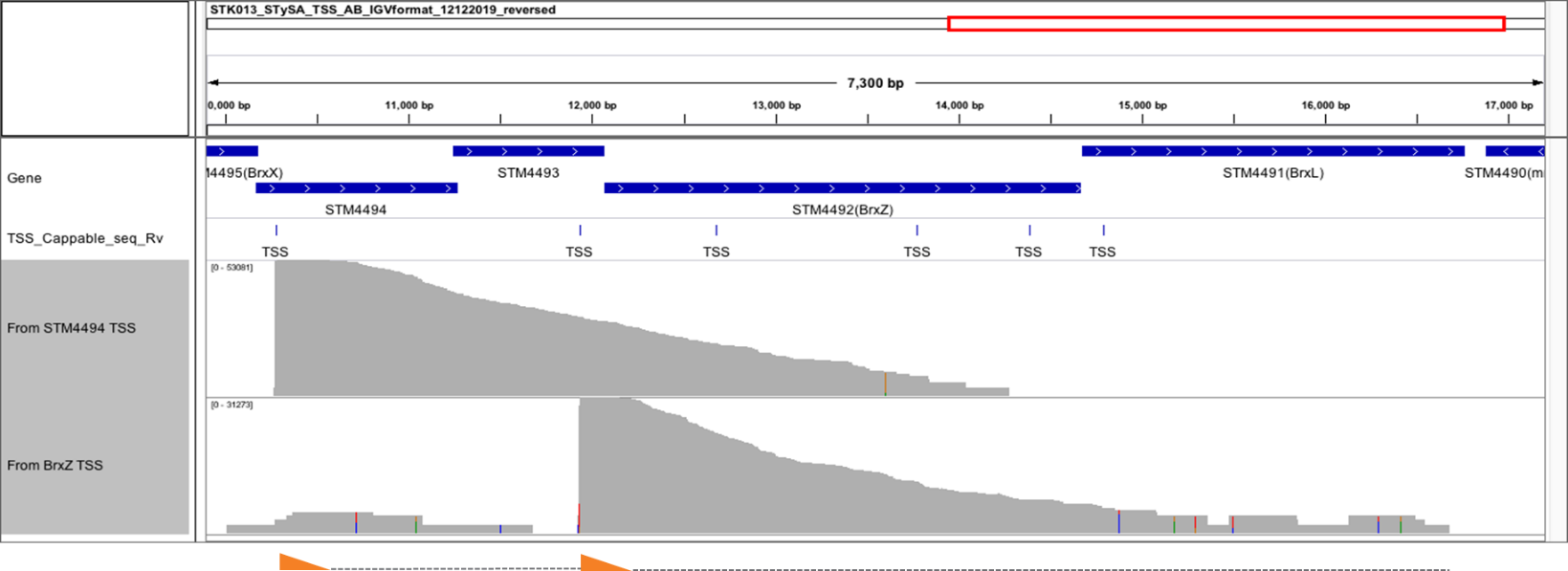

Supplement: S8 File — Four figures: Sample isolation strategy and transcripts probed for brxA, ATPase, and DUF4435 TSS visualized with IGV sequence read alignment screenshots at different magnifications. (DOCX) [file pgen.1009943.s011.docx]
