# Supplementary material for: Reassembling a cannon in the DNA defense arsenal: Genetics of StySA, a BREX phage exclusion system in Salmonella lab strains
Source: PLoS Genet. 2022 Apr 4;18(4):e1009943. doi: 10.1371/journal.pgen.1009943 (PMC9009780; doi:10.1371/journal.pgen.1009943)
Supplement: S9 File — (DOCX) [file pgen.1009943.s012.docx]

**A. ER3625 annotation protocol used in analysis of RNAseq output**

**For File S5**

This protocol yields columns in order of Locus_tag with associated functional information for a single sequence. Columns are added to RNAseq files with Locus_tag ordered data to inform interpretation of otherwise anonymous Locus_ID.

Only CDS features from ER3625 and its plasmid are included in the RNAseq data and in the annotation addition to it represented by this protocol.

Source files:

DNA sequence with annotation: ER3625 Genbank CP067091 (chromosome) and CP067091 (plasmid)

RNASeq transcripts per feature: output of htseq (v. 0.9.1) as described in Material and Methods

Differential expression data relative to ER3625

Output file used for analysis of RNAseq:

"S5_File RNAseq_genome_hits",--sheet 3, "ER3625 annot w_reg and plasmid"

Rows 4506-4618 are pSLT source

Additional destination for annotations is RNAseq data in S5 File describing the intersections of differentially-expressed features shared by defined strain sets.

Sort by locus_tag for attaching annotations

Three sets of data transfer:

1. NCBI chromosome annotations

2. user-defined "region" annotations

3. NCBI plasmid annotations.

1. In Geneious:

Geneious Prime 2021.1.1
Build 2021-03-12 13:25

Chromosome CP067091 sequence:

Export CDS Locus_tag annotation file

- select CDS annotations only
- create table with (in order)
  - locus_tag (this will be redundant for checking purpose in combined table)
  - name
  - minimum (original)
  - maximum (original)
  - direction
  - Length
  - Product
  - Note
- sort on locus_tag
- export as .csv
- Name the file as "Annotation table from strain X" or some such.

On computer:

- Open .csv file with annotations
- Save as .xlsx version
- Make sure the headers are correct and locus_tags in order
- Open RNAseq data file
- Make a copy of the data sheet, rename "annot" or some such
- Add header titles to additional columns: Locus_tag, name, minimum, maximum, direction, length, product, Note. These will be the columns to be replaced by the same columns from the annotation file.
- Copy columns from annotation file
- Paste into the new RNAseq data sheet.
- Check for correlation of locus_tag in the first column of RNAseq data and the new column just added

For convenience and legibility, replace "Derived by automated computational analysis using gene prediction method: Protein Homology." in the "Note" column with blank space, to allow reading of significant notes. Added a column for "region"

2. To generate "region" identifications such as prophages and genome islands.

Modify the NCBI-annotated sequence file: Geneious file "ER3625 copy with regions tracked Salmonella enterica subsp. enterica serovar Typhimurium - CP067091"

In Geneious:

- Define annotation type "Landmark" chromosomal segments of interest
- Create a track named "Regional Features"
- Identify limits of regions with Mauve alignments of CP067091 to segments extracted from NCBI sequence AE006468
  - Fels-1 "Source" annotation "Salmonella phage Fels-1"
  - Gifsy-2 "Source" annotation "Salmonella phage Gifsy-2"
  - Gifsy-1 "Source" annotation "Salmonella phage Gifsy-1"
- Identify limits of other regions
  - H1 Abony segment reported in Zaworski et al {Zaworski, 2021 #9374}
  - H2 Abony segment reported in Zaworski et al {Zaworski, 2021 #9374}
  - BREX - StySA defined here
  - ICR defined Sibley et al. {Sibley, 2004 #3810}
- Select Track "landmark" in annotations screen
- Export table
- Name
- Type
- minimum
- maximum
- length
- direction
- Track name
- Sort on Minimum
- Export as .csv

Exported "ER3625 regions tracked Salmonella enterica subsp enterica serovar Typhimurium - CP067091"

3. To generate "plasmid" annotations

Exported plasmid annotations from CP067092 "Salmonella enterica subsp. enterica serovar Typhimurium strain ER3625 plasmid pSLT, complete sequence"

Copied rows 2:114 (not header)

Pasted at the end

Replaced "Derived by automated computational analysis using gene prediction method: Protein Homology." with blank

added "pSLT" in "Region" column

Rows 4506-4618 are pSLT source

4. To assess the effect of an allele shared among strains:

- Used DESeq results obtained as described in Materials and Methods.
- List of features meeting the cutoff was generated and ordered by Locus_tag for each strain
- Applied the R command "Intersect" to lists pertaining to sets of strains with shared properties of interest
- These features were listed by strain set on sheet "Category summary" of "S5 File RNAseq_genome_hits.xlsx"
- Sets were
- ∆*brxL*
- ∆*brxB*
- Strains that were refractory to phage L infection
- On sheet "Category key", combined feature lists for all sets were deduplicated and sorted in chromosomal order
- Colum A:I for each Locus_tag Row was copied from sheet "ER3625 annot w_reg and plasmid" of "S5 File RNAseq_genome_hits.xlsx" and added to sheet "Category key"
- Locus_tags shared between strain sets on sheet "category summary" were manually color-coded by functional shorthand.
- Locus_tags unique to a strain set were evaluated for significance to the phenotypes displayed by the set.
